# The Ever-Expanding *Pseudomonas* Genus: Description of 43 New Species and Partition of the *Pseudomonas putida* Group

**DOI:** 10.3390/microorganisms9081766

**Published:** 2021-08-18

**Authors:** Léa Girard, Cédric Lood, Monica Höfte, Peter Vandamme, Hassan Rokni-Zadeh, Vera van Noort, Rob Lavigne, René De Mot

**Affiliations:** 1 Centre of Microbial and Plant Genetics, Faculty of Bioscience Engineering, KU Leuven, Kasteelpark Arenberg 20, 3001 Leuven, Belgium; lea.girard@kuleuven.be (L.G.); cedric.lood@kuleuven.be (C.L.); vera.vannoort@kuleuven.be (V.v.N.); 2 Department of Biosystems, Laboratory of Gene Technology, KU Leuven, Kasteelpark Arenberg 21, 3001 Leuven, Belgium; 3 Department of Plants and Crops, Laboratory of Phytopathology, Faculty of Bioscience Engineering, Ghent University, Coupure links 653, 9000 Ghent, Belgium; Monica.Hofte@ugent.be; 4 Laboratory of Microbiology, Department of Biochemistry and Microbiology, Faculty of Sciences, Ghent University, K. L. Ledeganckstraat 35, 9000 Ghent, Belgium; Peter.vandamme@ugent.be; 5 Zanjan Pharmaceutical Biotechnology Research Center, Zanjan University of Medical Sciences, Zanjan 45139-56184, Iran; hassan.roknizadeh@gmail.com; 6 Institute of Biology, Leiden University, Sylviusweg 72, 2333 Leiden, The Netherlands

**Keywords:** Pseudomonadaceae, long-read sequencing, hybrid assembly, taxonomy, phylogeny, Non-Ribosomal Peptide Synthetase (NRPS), Cyclic Lipopeptides (CLPs)

## Abstract

The genus *Pseudomonas* hosts an extensive genetic diversity and is one of the largest genera among Gram-negative bacteria. Type strains of *Pseudomonas* are well known to represent only a small fraction of this diversity and the number of available *Pseudomonas* genome sequences is increasing rapidly. Consequently, new *Pseudomonas* species are regularly reported and the number of species within the genus is constantly evolving. In this study, whole genome sequencing enabled us to define 43 new *Pseudomonas* species and provide an update of the *Pseudomonas* evolutionary and taxonomic relationships. Phylogenies based on the *rpoD* gene and whole genome sequences, including, respectively, 316 and 313 type strains of *Pseudomonas*, revealed sixteen groups of *Pseudomonas* and, together with the distribution of cyclic lipopeptide biosynthesis gene clusters, enabled the partitioning of the *P. putida* group into fifteen subgroups. Pairwise average nucleotide identities were calculated between type strains and a selection of 60 genomes of non-type strains of *Pseudomonas*. Forty-one strains were incorrectly assigned at the species level and among these, 19 strains were shown to represent an additional 13 new *Pseudomonas* species that remain to be formally classified. This work pinpoints the importance of correct taxonomic assignment and phylogenetic classification in order to perform integrative studies linking genetic diversity, lifestyle, and metabolic potential of *Pseudomonas* spp.

## 1. Introduction

During the past decade, the landscape of bacterial systematics has changed drastically [1]. Once dominated by a polyphasic approach including phenotypic characterization, DNA–DNA hybridization, and 16S rRNA gene sequencing, the age of microbial genomics and metagenomics has reshaped the foundation of prokaryotic species definition [2,3]. Although 16S rRNA phylogeny remains the most common tool to evaluate the diversity of mixed prokaryotic populations, estimating inter- and intra-species relatedness was traditionally facilitated by DNA-typing methods. For several years, Multi-Locus Sequence Analysis (MLSA) represented the most widely adopted methodology for bacterial systematics, and for the exploration of evolutionary relationships within specific families/genera [4,5,6,7]. The success of high throughput and affordable Whole Genome Sequencing (WGS) technologies has tremendously increased the number of publicly available genomes and, therefore, genome-to-genome comparisons, with the Average Nucleotide Identity (ANI) and digital DNA–DNA Hybridization (dDDH), have become today’s standards for species definition [1,8,9,10,11]. This genome-based elucidation of relatedness at the inter- and intra-species level is now encouraged and, at a larger scale, the creation of a Genome Taxonomy Database (GTDB) has allowed the bacterial taxonomy to be standardized [12,13].

According to GTDB, the Pseudomonadaceae family currently includes seven genera: *Azomonas*, *Azotobacter*, *Entomomonas*, *Oblitimonas*, *Pseudomonas*, *Thiopseudomonas*, and *Ventosimonas* (https://gtdb.ecogenomic.org/tree?r=f__Pseudomonadaceae, accessed on 10 July 2021). The genus *Pseudomonas* is the most complex, with 259 validly named species (List of Prokaryotic Names with Standing in Nomenclature (https://lpsn.dsmz.de/genus/pseudomonas, accessed on 10 August 2021), excluding subspecies and synonymous species. However, this number is constantly evolving, with over 30 new *Pseudomonas* species described between March 2020 and March 2021. Since the first descriptions of *Pseudomonas* species, which were based on morphological and phenotypical characteristics, several studies updated the taxonomy of *Pseudomonas* based on 16S rRNA gene sequence analysis [14]. This allowed the differentiation of the genus *Pseudomonas* from its sister genera, and also the definition of the three main *Pseudomonas* lineages, *P. pertucinogena*, *P. aeruginosa*, and *P. fluorescens* [6,15]. In a similar fashion, MLSA has guided the redefinition of prokaryotic species and has also impacted the phylogenomics and systematics of the genus *Pseudomonas* [4,6,16]. Indeed, the analysis based on four housekeeping genes (i.e., 16S rRNA, *gyrB*, *rpoB*, and *rpoD*) enabled the clarification of the *Pseudomonas* phylogeny by enhancing species delineation. This approach also proved to be a reliable tool for strain identification at the species level [4,6]. We recently demonstrated that the *rpoD* gene sequence alone provides a strong and low-cost alternative, particularly in the case of taxonomic affiliation of large batches of environmental *Pseudomonas* isolates [17].

*Pseudomonas* are motile, non-spore forming, Gram-negative rods belonging to the *Gammaproteobacteria*. *Pseudomonas* species are able to colonize and thrive in a wide range of ecological niches (e.g., soil, water, and plants, associated with higher organisms) [18]. In addition to the well-known human pathogen *P. aeruginosa*, other *Pseudomonas* species induce diseases in plants, fish, insects, or other animals [19,20,21]. In contrast, a large majority of *Pseudomonas* species are commensals but can also be used as bioremediation, biostimulation, and biocontrol agents [22,23]. *Pseudomonas* are ubiquitous bacteria that are often identified as fundamental components of bacterial communities and thus play essential ecological functions in the environment [24,25,26]. Furthermore, *Pseudomonas* are outstanding producers of bioactive secondary metabolites that often support their eclectic lifestyle (e.g., iron scavenging, swarming motility, biofilm formation, pathogenicity, cooperation, or antagonism) [27,28]. The link between secondary metabolites and *Pseudomonas* taxonomy has already been made through pyoverdines, a class of pigments used for a long time as a specific marker of classification [18]. *Pseudomonas* cyclic lipopeptides (CLPs), having a broad antimicrobial activity profile and anti-proliferative properties, have gained the attention of researchers due to their promising application potential [29]. CLP production is widespread within the genus *Pseudomonas*, and relationships between CLP diversity and *Pseudomonas* taxonomy were recently highlighted [30,31]. CLP producers tend to be grouped by CLP family and confined to specific groups or subgroups of *Pseudomonas.* Nonetheless, exceptions occur within the *P. putida* group, which hosts a large diversity of CLP producers from diverse families (i.e., Xantholysin, Entolysin, Putisolvin, and Viscosin families) [30,31].

In this study, we report 43 new *Pseudomonas* species and use a combination of Nanopore and Illumina sequencing to provide high quality genomes. Through the genome analysis of these new species, together with type strains of *Pseudomonas*, we provide an update of the *Pseudomonas* phylogeny based on a set of 1508 core orthogroup sequences and another based on the *rpoD* gene. We used nucleotide identities based on the *rpoD* gene and whole genome comparisons to reassign, respectively, 82 and 41 non-type strains of *Pseudomonas* to known and newly described *Pseudomonas* species. A large majority of the new species were affiliated to the *P. putida* group, increasing species numbers from 35 to 51. We thus explored genetic diversity within the *P. putida* group in a greater depth and mapped, on an expanded phylogeny of the group, the presence of Biosynthetic Gene Clusters (BGCs) for the production of CLPs.

## 2. Materials and Methods

### 2.1. Pseudomonas Strains

In this study we used 273 known type strains of *Pseudomonas*, including validly published species and recently published species still lacking taxonomic status (https://lpsn.dsmz.de/genus/pseudomonas, accessed on 10 August 2021). Only 270 type strains of *Pseudomonas* were used for genome analysis because no genome sequences were available for three of these type strains. Eleven type strains of other genera within the Pseudomonadaceae and *Cellvibrio japonicus* were used for phylogenetic analyses (Figure S5). The list of type strains, including their culture collection codes and accession numbers (i.e., *rpoD* and whole genome sequences) is provided in Table S1.

We also used 47 strains from our collection of environmental *Pseudomonas* isolates to describe 43 new *Pseudomonas* species (the type strains of newly described species are highlighted in bold; Table S2). These 47 isolates were deposited in two culture collections (i.e., Belgian Co-ordinated Collections of Micro-organisms (BCCM/LMG) and Collection Française de Bactéries associées aux Plantes (CFBP)), and their phenotypic profiles were obtained using the Biolog GEN III MicroPlate (BIOLOG, Hayward, CA, USA) according to the manufacturer’s instructions (Table S3). To avoid species description based on single strains, the *rpoD* sequences of these 43 new type strains of *Pseudomonas* were then used as a query to search for additional strains using BlastN with default parameters (https://blast.ncbi.nlm.nih.gov/Blast.cgi?PAGE_TYPE=BlastSearch; accessed on 10 August 2021, Tables S4 and S5). We previously defined a cutoff value of 98% nucleotide identity to differentiate strains at the species level but also revealed some inconsistent species affiliations [17]. To avoid any misidentification due to the use of the *rpoD* gene, we only considered hits with 100% identity, or lower if a genome was available and thus allowed validation by ANIb calculation (> 96.5%, Tables S4 and S5). We thus used a total of 82 non-type strains of *Pseudomonas* (Table S4), including 29 with whole genome sequences (Table S5).

Finally, a set of 122 strains, including type and non-type strains of *Pseudomonas,* was specifically used for the *P. putida* group phylogenetic and genomic analyses (Table S6).

### 2.2. Genome Sequencing, Assembly, and Functional Annotation

We recently highlighted the high discriminative power of the *rpoD* gene as a reliable tool for the identification of environmental *Pseudomonas* isolates [17]. In the same study, we released draft genome sequences of 55 environmental *Pseudomonas* isolates and *rpoD* gene analysis together with whole genome comparisons allowed us to highlight the presence of 30 new *Pseudomonas* species (*Pseudomonas* #5 [17], with strains SWRI59, SWRI68, and SWRI77, was later identified as *P. capeferrum*). We applied the same methodology to an expanded set of *Pseudomonas* isolates and identified 17 additional new species. To provide high-quality genomes for the type strains of these 43 new species, we combined Illumina and Nanopore sequencing [32]. An overview of the different sequencing methodologies used for the entire set of strains is shown in Table S2. We controlled the quality of the Illumina reads with FastQC v0.11.9 and used Trimmomatic v0.38 [33] for adapter clipping, quality trimming (LEADING:3 TRAILING:3 SLIDINGWINDOW:4.15), and filtering on length (>50 bp). The quality of the Nanopore reads was assessed with Nanoplot v1.28.2 [34] and we used Porechop v0.2.4 (https://github.com/rrwick/Porechop, accessed on 8 June 2021) for barcode clipping, in addition to NanoFilt v2.6 [34] to filter quality (Q > 8) and length (>500 bp). The genomes were assembled using Unicycler v0.4.8 [35] with default options and the quality of their assemblies was assessed using QUAST v5.1 [36]. The functional annotation was undertaken with the NCBI Prokaryotic Genome Annotation Pipeline [37].

### 2.3. Taxonomic Affiliations and Phylogenetic Analyses

To define the new species and confirm *rpoD*-based affiliations, Average Nucleotide Identity (ANI) values were calculated using PYANI v0.2.10 [38] with default parameters, and the ANIb method (Table S7) with a cutoff value of 96.5% [17,18]. In the case of ANIb values considered as ambiguous (i.e., between 95 and 96.5%) we calculated digital DNA–DNA Hybridization (dDDH) using the Genome-to-Genome Distance Calculator (GGDC; https://www.dsmz.de/services/online-tools/genome-to-genome-distance-calculator-ggdc, accessed on 8 June 2021).

**Figure 1:**
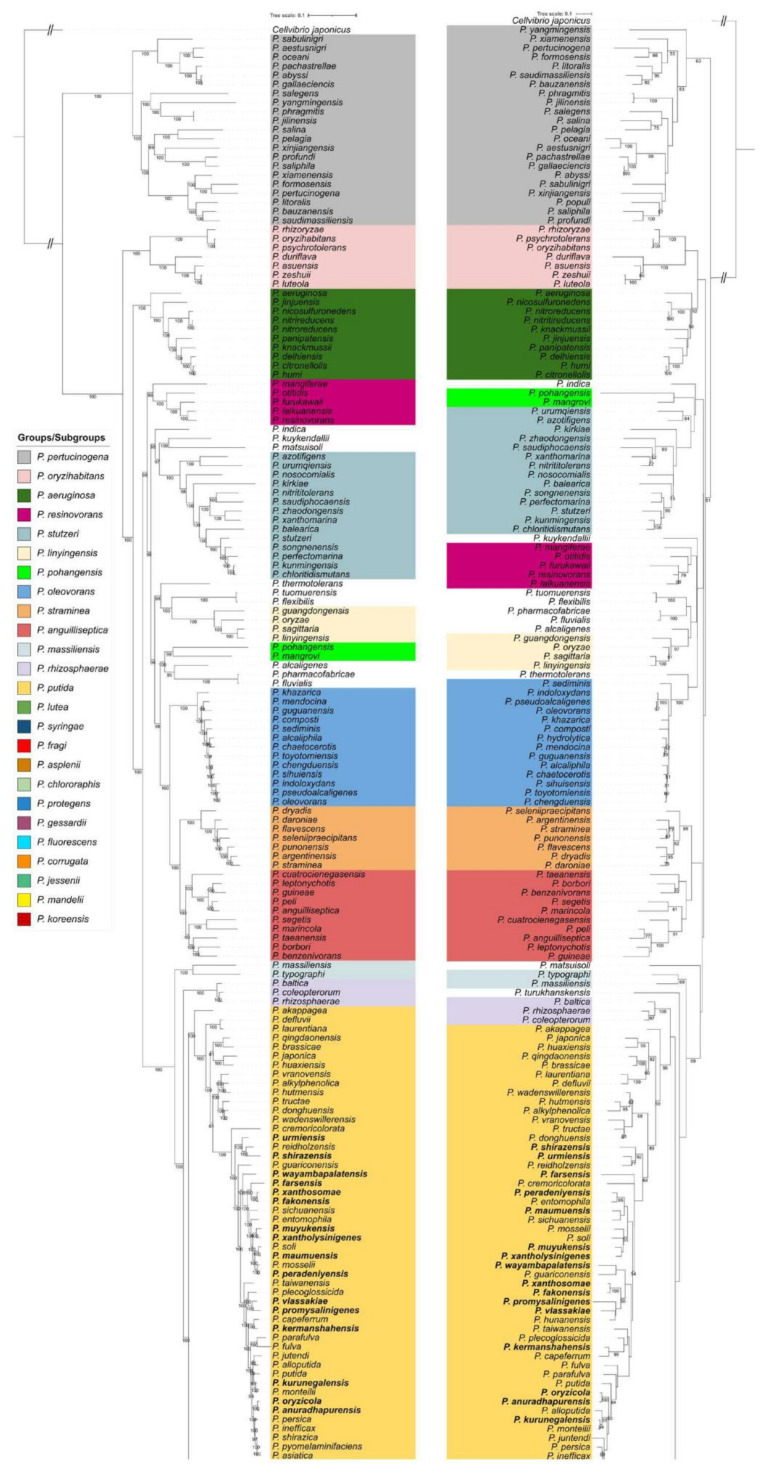

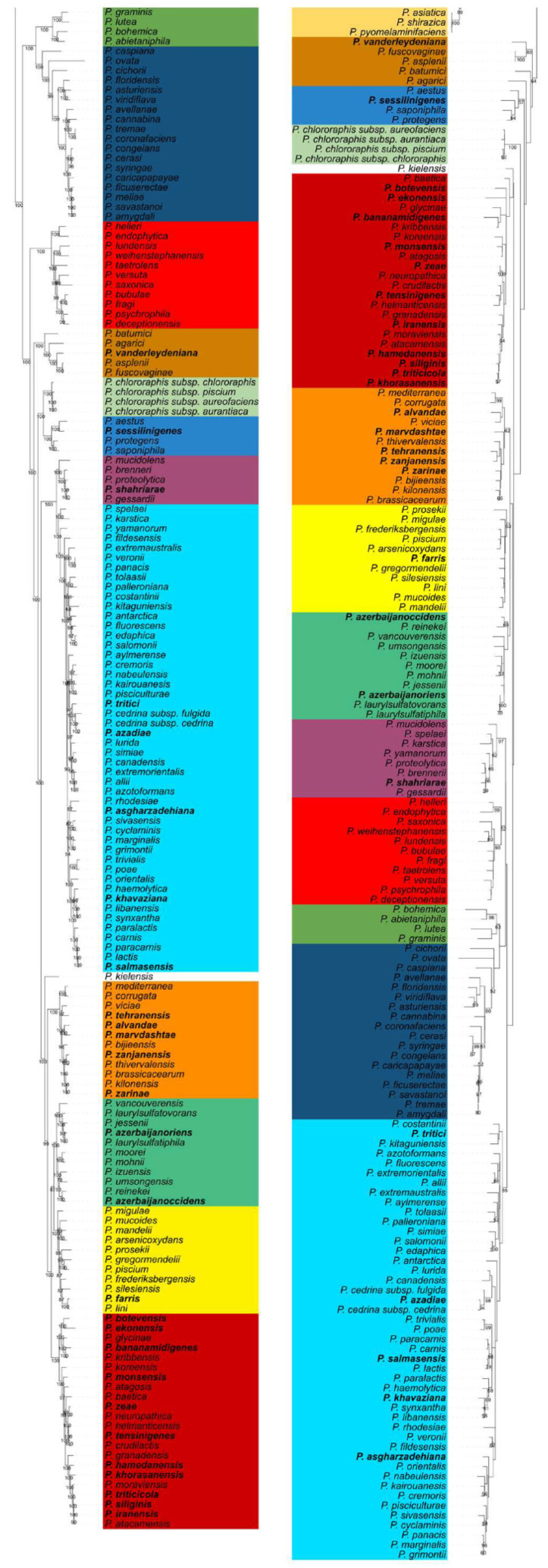
Phylogenetic tree based on 1508 core orthogroups using IQ-TREE with automatic model selection and 1000 ultrafast bootstraps (**left**) and the *rpoD* gene (**right**); maximum likelihood tree, GTR + G+I model (MEGA-X)) including, respectively, 313 and 316, type strains of *Pseudomonas*. Bootstrap values were calculated based on 1000 replications. Type strains of newly described species are highlighted in bold. *Cellvibrio japonicus* is used as the outgroup.

The evolutionary relationships between newly described and previously known type strains of *Pseudomonas* were assessed using *rpoD* and whole genome phylogenies. The *rpoD*-based phylogenies were conducted as previously described using MEGA-X (Figure 1, right) [17]. The corresponding similarity matrix, based on a 650 bp fragment of the *rpoD* gene, including 316 type strains of *Pseudomonas* (273 known and 43 newly described species), was generated (Table S8). The phylogenetic trees based on whole genomes were inferred with IQ-TREE v1.6.12 [39] with automatic model selection and 1.000 ultrafast bootstraps (UF-Boot) using an alignment of 1508 (genus phylogeny; Figure S1, left) and 2570 (*P. putida* group phylogeny; Figure S5) core orthogroup sequences that were delineated with the SCARAP pipeline (https://github.com/SWittouck/SCARAP, accessed on 8 June 2021) [40].

**Figure 2:**
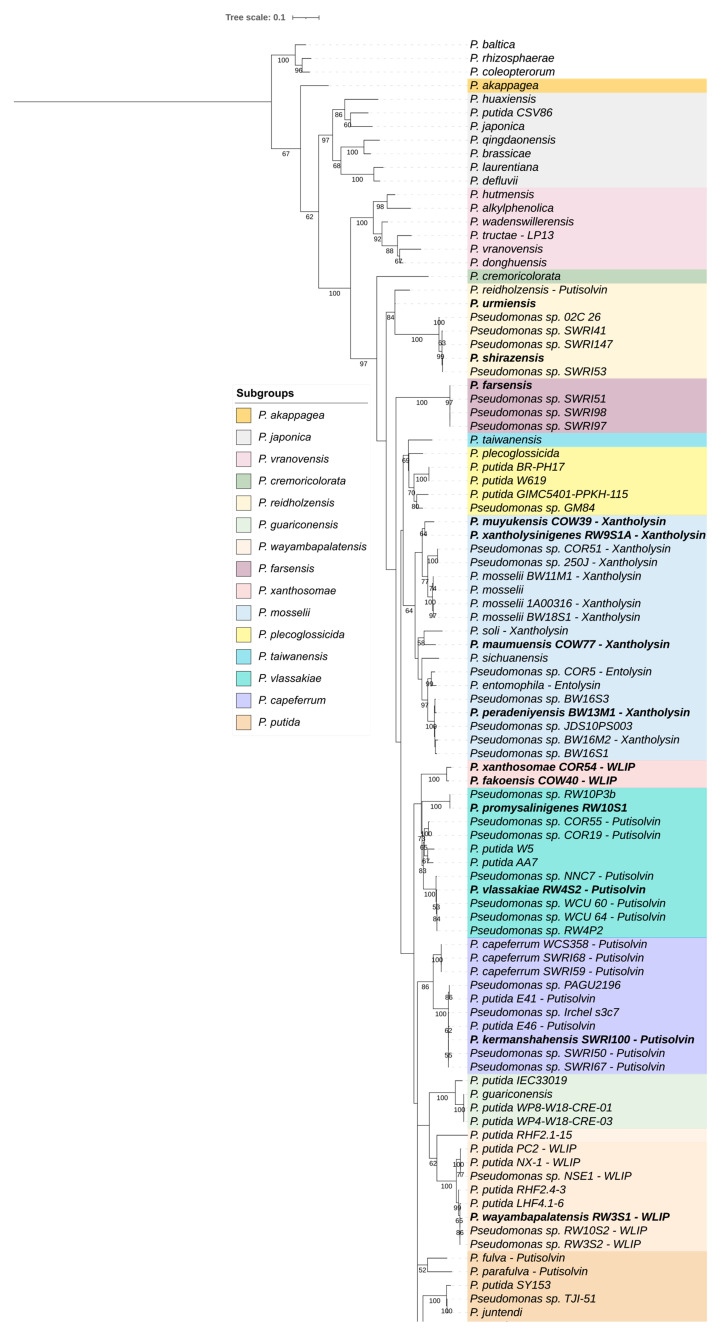

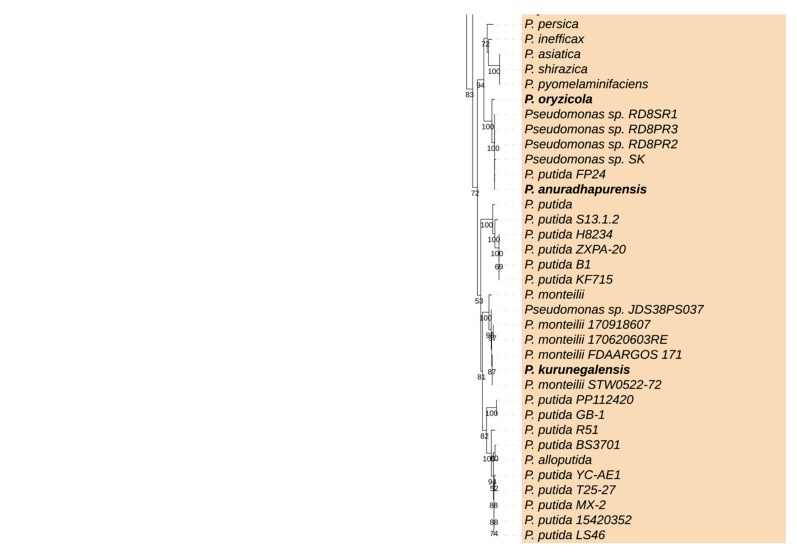
Phylogenetic tree of the *P. putida* group based on the *rpoD* gene of 122 *Pseudomonas* strains (Table S6). All the strains included in this analysis, together with their accession numbers and the output of the prospection for CLP BGCs, are detailed in Table S6. The maximum likelihood phylogenetic tree was constructed using the GTR + G+I model (MEGA-X). Bootstrap values were calculated based on 1000 replications and only bootstrap values higher than 50% are indicated. Type strains of newly described species are highlighted in bold. The *P. rhizosphaerae* group is used as the outgroup. The corresponding tree based on whole genome sequences is shown in Figure S5.

Several new phylogenetic groups (G) and subgroups (SG) were delineated based on branch length, grouping, and bootstrap values on both *rpoD* and whole genome phylogenies (Table 1 and Table 2, Figure 1, Figure 2, and Figure S5). The new groups and subgroups were named after the first species described in a group or subgroup.

### 2.4. Cyclic Lipopeptide (CLP) NRPS Analysis

The *P. putida* group was previously highlighted to include CLP producers from the Viscosin (WLIP producers), Putisolvin, Entolysin, and Xantholysin families [30,31]. Among the 16 type strains of the newly described species belonging to the *P. putida* group, 4 were already described as CLP producers (WLIP and Xantholysin producers) [30,31]. Consequently, all strains belonging to the *P. putida* group (Table S6), with available genome sequences, were subjected to an antiSMASH analysis (antiSMASH 6.0) [41]. Positive hits were then inspected manually to confirm the typical features of *Pseudomonas* CLP Non-Ribosomal Peptide Synthetase (NRPS) clusters (i.e., the presence of tandem TE-domains and the absence of epimerization domains) and synteny (i.e., number of modules and their distribution along the encoded NRPSs), all based on previously described CLP NRPS gene cluster annotations [42,43]. All known and newly identified strains carrying CLP BGCs, together with their affiliation to CLP families and the accession numbers of their NRPS genes, are presented in Table S6. The phylogenetic relationship between known and newly identified CLP producers was assessed, by family, based on concatenated NRPS amino acid sequences (Figures S2–S4).

## 3. Results and Discussion

### 3.1. Defining New Pseudomonas Species

In a recent study, we performed *rpoD*-based identifications which allowed us to identify 31 new *Pseudomonas* species [17]. In the same study, three strains were incorrectly identified as representative strains of a new species (i.e., *Pseudomonas* #5, SWRI59, SWRI68, and SWRI77) but subsequently identified as *P. capeferrum* strains. Further *rpoD*-based identifications enabled us to identify 17 additional *Pseudomonas* species. Four strains, namely, SWRI22, OE 28.3, SWRI76, and CMR5c, were first assessed as new species but were later assigned to newly published *Pseudomonas* species (i.e., #29 *P. carnis*, #30 *P. edaphica*, #31 *P. atacamensis*, and #45 *P. aestus*; Table S7). Finally, a total of 43 new *Pseudomonas* species could be defined (Appendix A) and the result of their phenotypic profiling, together with assigned culture collection numbers, are presented in Table S3. Hybrid assemblies of the genomes resulted in 22 closed genomes and 18 draft genomes with improved contiguity. Due to technical issues, we have not been able to increase the quality of the draft genomes of strains BW11P2, COW3, and SWRI196. To avoid the proposal of new species based on single strains, the *rpoD* sequences of the 43 new species were used as queries to search for additional strains. We therefore reassigned 82 *Pseudomonas* strains, including 29 with whole genome sequences, available through GenBank (Tables S1 and S2). Finally, ANIb values were calculated between a total of 346 *Pseudomonas* species (270 type strains and 76 (47 + 29) *Pseudomonas* strains affiliated to new species), and allowed us to confirm these affiliations and the presence of 43 new *Pseudomonas* species (Table S7). The phylogenetic position of the 43 type strains is shown in Figure 1 and their distribution within the different groups of *Pseudomonas* is detailed in Table 1. All of the new species are clustering within the *P. fluorescens* (*n* = 27) and *P. putida* (*n* = 16) groups. We amended the existing subgroups of *P. fluorescens* as follows: *P. asplenii* (inclusion of *P. vanderleydeniana*), *P. corrugata* (inclusion of *P. alvandae, P. marvdashtae, P. tehranensis, P. zanjanensis* and *P. zarinae*), *P. fluorescens* (inclusion of *P. asgharzadehiana*, *P. azadiae*, *P. khavaziana*, *P. salmasensis* and *P. tritici*), *P. gessardii* (inclusion of *P. shahriarae*), *P. jessenii* (inclusion of *P. asgharzadehiana* and *P. azerbaijanoccidens*), *P. koreensis* (inclusion of *P. bananamidigenes*, *P. botevensis*, *P. ekonensis*, *P. hamedanensis*, *P. iranensis*, *P. khorasanensis*, *P. monsensis*, *P. siliginis*, *P. tensinigenes*, *P. triticicola* and *P. zeae*), *P. mandelii* (inclusion of *P. farris*), *P. protegens* (inclusion of *P. sessiligenes*) (Table 1). The remaining sixteen new species allowed the partitioning of the *P. putida* group into fifteen subgroups, as described in Section 3.3.

### 3.2. Comparison of Whole Genome and rpoD-based Phylogenies

The phylogenetic relationships between known and newly described type strains of *Pseudomonas* are presented in Figure 1, respectively, the whole genome, based on 1508 core orthogroups, and the *rpoD*-based phylogenies. The phylogenies include 273 type strains of *Pseudomonas* species (270 for the whole genome phylogeny) and 43 type strains of the newly described *Pseudomonas* species. Three type strains of *Pseudomonas* were excluded from the analysis: (1) *P. hydrolytica*, with an abnormally long genome (10.4 Mbp)); and (2) *P. hussainii* and *P. caeni*, harboring short genomes (respectively, 3.68 and 3.03 Mbp) and clustering with members of other genera within the Pseudomonadaceae [4,6,17]. We suspect that the latter two are not *Pseudomonas* species and a dedicated study needs to clarify the taxonomy of other genera within the Pseudomonadaceae family.

Indeed, *P. caeni* gained the attention of Hesse and colleagues [18] due to its unusual genomic features, and is already displayed as *Thiopseudomonas caeni* in the GTDB (https://gtdb.ecogenomic.org/tree?r=f__Pseudomonadaceae, accessed on 10 July 2021). A tree of the Pseudomonadaceae family, including *P. hussainii* and *P. caeni* (*T. caeni*), in addition to all type strains of the *Azomonas*, *Azotobacter*, *Entomomonas*, *Oblitimonas*, *Pseudomonas*, *Thiopseudomonas*, and *Ventosimonas* genera, is shown in Figure S1.

The thirteen groups of *Pseudomonas* previously identified in several studies (i.e., *P. pertucinogena, P. oryzihabitans, P. aeruginosa, P. resinovorans, P. stutzeri, P. linyingensis, P. oleovorans, P. straminea, P. anguilliseptica, P. putida, P. lutea, P. syringae*, and *P. fluorescens*) are all well supported in both trees [4,6,17,18]. In addition to these thirteen groups, three new groups, namely, *P. pohangensis, P. massiliensis*, and *P. rhizosphaerae*, were identified based on branch length and the strong bootstrap support values separating them from the neighboring groups (Figure 1). Furthermore, as previously observed, ten species are scattered across the tree and represent orphan groups currently formed by only one species (Figure 1). An overview of all known and newly proposed groups is summarized in Table 1.

Overall, both trees are highly consistent in topology, although the tree inferred by whole genome analysis is supported by stronger bootstrap values. Two main differences can still be highlighted: (1) the position of the *P. syringae* and *P. lutea* group, clustering inside the *P. fluorescens* group in the *rpoD*-based tree; and (2) the position of *P. karstica*, *P. spelaei*, and *P. yamanorum*, clustering within the *P. gessardii* subgroup in *rpoD* and MLSA phylogenies [4,6,17], whereas in phylogenies based on whole genome analysis, they cluster within the *P. fluorescens* subgroup ([18] and Figure 1).

### 3.3. Genomic Diversity within the P. putida Group

#### 3.3.1. Identification and Reassignment at the Species Level

Several studies have revealed inconsistencies within public databases in which genomes of *Pseudomonas* are not identified (*Pseudomonas* sp.) or incorrectly assigned at the species level [4,44,45]. Within the *P. putida* group, a huge number of strains are incorrectly assigned to *P. putida* [4,44]. Here, we propose to update the *P. putida* group with 16 new *Pseudomonas* species and tentatively reassign 44 non-type strains of *Pseudomonas* (Table 3). A total of 25 strains are affiliated to known and newly described species (*P. shirazensis* (*n* = 1), *P. guariconensis* (*n* = 2), *P. wayambapalatensis* (*n* = 2)*, P. farsensis* (*n* = 1), *P. peradeniyensis* (*n* = 1), *P. capeferrum* (*n* = 2), *P. kermanshahensis* (*n* = 4), *P. juntendi* (*n* = 2), *P. alloputida* (*n* = 6), and *P. kurunegalensis* (*n* = 4)), and the remaining 19 strains represent an additional 13 new species. As previously observed for the genus *Pseudomonas*, these results confirm the fact that type strains still represent a small fraction of the genomic diversity within the *P. putida* group.

#### 3.3.2. Distribution of CLP biosynthesis Gene Clusters

CLPs are specialized metabolites that often support important ecological functions including cooperation, phytopathogenicity, or antagonism [29,43,46]. CLPs consist of a fatty acid tail attached to a cyclized oligopeptide and are synthesized by NRPSs [29,42]. The modularity of these enzymes enables *Pseudomonas* strains to produce a wide diversity of CLPs, resulting in their classification in several families [28,29,42]. The relationship between CLP diversity and *Pseudomonas* taxonomy was recently highlighted, and it was demonstrated that certain CLP families were exclusive to specific subgroups of *P. fluorescens* [30,31,43]. In contrast, the *P. putida* group was demonstrated to host CLP producers from different families [30,31]. CLP production is widespread within the *P. putida* group and different type strains (i.e., *P. capeferrum*, *P. entomophila*, and *P. soli*) and many non-type strains (e.g., RW10S2, PCL1445, BW11M1, 250J, COR5, COW10, COR19, COR51; Table S7) were formerly characterized as producers of CLPs from the Viscosin (WLIP producers), Putisolvin, Entolysin, and Xantholysin families [30,31,46,47,48,49,50,51,52,53,54,55,56]. Among the 16 type strains of the newly described species, four were previously described as CLP producers: two WLIP producers, *P. fakonensis* COW40 and *P. xanthosomae* COR54; and two xantholysin producers, *P. maumuensis* COW77 and *P. muyukensis* COW39 (Table S6) [30,31]. We therefore searched for CLP NRPSs in a selection of *Pseudomonas* genomes, including all type strains belonging to the *P. putida* group (*n* = 51) and the 44 genomes of non-type strains presented in Table 3. About 65% of the strains (i.e., 34 of 51 type strains; 28 of 44 non-type strains) did not carry CLP NRPSs in their genomes (Table S6). Our analysis revealed the presence of NRPSs from the Viscosin family (WLIP-like NRPSs) in the genomes of two strains (*P. wayambapalatensis* RW3S1^T^ and RW3S2); from the Putisolvin family in five type strains (*P. fulva*, *P. kermanshahensis*, *P. parafulva*, *P. reidholzensis*, and *P. vlassakiae*) and five non-type strains (*P. capeferrum* SWRI59 and SWRI68 and *P. kermanshahensis* SWRI67, SWRI50, E46); and from the Xantholysin family in two type strains (*P. peradeniyensis* and *P. xantholysinigenes*) and two non-type strains (*P. mosselii* BW18S1 and *P. peradeniyensis* BW16M2) (Figure 2 and Table S6). The poor genome quality of two type strains, namely, *P. brassicae* and *P. juntendi*, revealed the presence of NRPS gene fragments coding for tandem thioesterase (TE) domains. Tandem TE domains are specific to *Pseudomonas* CLP NRPS; therefore, these results indicate *P. brassicae* and *P. juntendi* carry CLP NRPS genes and most likely produce CLPs. Further analyses, chemical characterization, and/or a hybrid assembly based on long read sequencing and Illumina sequencing are needed to identify the CLPs. We previously highlighted, in the type strains of *P. asplenii* and *P. fucovaginae*, a NRPS system predicted to assemble a lipotridecapeptide (LP-13) but this metabolite still awaits chemical and functional characterization [43]. A putative LP-13 biosynthesis gene cluster is also present in the genome of *P. tructae*. Oni and colleagues also reported the presence of a new CLP (N8, 17:8, 17 amino acids, of which 8 are in the macrocycle) within the *P. putida* group [30,31]. Altogether, these results highlight a wide diversity of CLP producers from known, and yet to be described new, CLP families within the *P. putida* group.

#### 3.3.3. Partitioning of the *P. putida* Group

To present an integrated approach linking the genetic diversity and the metabolic potential of *Pseudomonas* species, we mapped the presence of CLP biosynthesis gene clusters on an extended phylogeny of the *P. putida* group (Figure 2 and Figure S5). As shown in Figure 1, the *P. putida* group is composed of several subgroups (Figure 1). The extended phylogeny allowed us to define 15 subgroups, *P. japonica* (*n* = 6), *P. vranovensis* (*n* = 6), *P. reidholzensis* (*n* = 3), *P. xanthosomae* (*n* = 2), *P. mosselii* (*n* = 8), *P. vlassakiae* (*n* = 3), *P. capeferrum* (*n* = 2), and *P. putida* (*n* = 14), including seven orphan subgroups (*P. akappagea*, *P. cremoricolorata*, *P. guariconensis*, *P. wayambapalatensis*, *P. farsensis*, *P. taiwanensis*, and *P. plecoglossicida*). The distribution of all type strains in the 15 subgroups is detailed in Table 1. Among the 44 non-type strains used in Section 3.3.1, 19 were highlighted to represent 13 new species distributed in seven subgroups: *P. japonica* (*n* = 1), *P. guariconensis* (*n* = 1), *P. wayambapalatensis* (*n* = 1), *P. mosselii* (*n* = 1), *P. plecoglossicida* (*n* = 3), *P. vlassakiae* (*n* = 2), and *P. putida* (*n* = 4) (Table 3). These additions to the *P. putida* phylogenies allowed us to seize a small portion of the genomic diversity among environmental *Pseudomonas* strains, but also to pinpoint the immediate growing potential of the newly defined subgroups. We observed that, in both *rpoD* and whole genome phylogenies, the distribution of CLP biosynthesis gene clusters was associated with this phylogenetic subgrouping.

All xantholysin and entolysin producers were grouped within the *P. mosselii* subgroup, putisolvin producers were clustered in four subgroups (i.e., *P. putida*, *P. reidholzensis*, *P. vlassakiae*, and *P. capeferrum*), and WLIP producers were distributed over two subgroups (*P. xanthosomae* and *P. wayambapalatensis*). Moreover, the phylogenies based on concatenated NRPS amino acid sequences (Xantholysin/Entolysin families, Figure S2; Putisolvin family, Figure S3; and WLIP producers, Figure S4) revealed different clusters that perfectly match the distribution of CLP producers within the different subgroups. These results demonstrate that the *rpoD* gene allows both the identification of *Pseudomonas* isolates and the construction of robust phylogenies, providing information about the affiliation of producers to CLP families.

The strong congruence between the phylogenetic tree based on the NRPS sequences and the rpo*D*- and whole genome-based phylogenies indicates that CLP biosynthesis genes have largely evolved in accordance with the evolutionary history of *Pseudomonas* species within the *P. putida* group. However, *P. reidholzensis* carries a putisolvin biosynthetic gene cluster that is absent from the genome of the closely related species. Furthermore, CLP producers from the Viscosin family, including WLIP producers, are predominantly found within the *P. fluorescens* group [30,31,57], with the exception of the two subclusters of WLIP producers present in the *P. putida* group. Altogether, these observations indicate that *Pseudomonas* CLP NRPS clusters have a complex evolutionary history probably involving both vertical and horizontal gene transfer.

## 4. Conclusions

Our update of the genus with 43 new species together with our analysis of 313 genomes of type strains allowed us to propose a robust revised phylogeny of the *Pseudomonas* spp. This study aimed to fill the gap between the currently named species and the real genomic diversity within the genus *Pseudomonas*. Additional work is needed to complete this task and genome-based standards for species definition should be favored over highly variable phenotypic tests for publication. Our study validated the use of the *rpoD* gene for species identification, and for the study of the evolutionary relationships within the genus *Pseudomonas*. Furthermore, *rpoD*-based phylogenies can also be highly useful to specifically prospect for CLP biosynthesis gene clusters and affiliation of producers to known CLP families. Finally, the use of genomic sequences appears to be essential to reveal the ecological and metabolic potential of *Pseudomonas* spp.

## Figures and Tables

**Table 1:** Newly proposed and emended groups of *Pseudomonas*. Newly described *Pseudomonas* species are highlighted in bold. ANIb and *rpoD* identity ranges were extracted from Tables S7 and S8).

Groups/Subgroups	Species/Subspecies	Total No. of Species/ Subspecies	*rpoD* Identity (%) ^1^	ANIb (%) ^1^
Existing Groups				
***P. aeruginosa G***	*P. aeruginosa, P. citronellolis, P. delhiensis, P. humi, P. jinjuensis, P. knackmussii, P. nicosulfuronedens, P. nitritireducens, P. nitroreducens, P. panipatensis*	10	77.85–95.98	80.57–94.48
***P. anguilliseptica G***	*P. anguilliseptica, P. benzenivorans, P. borbori, P. cuatrocienegasensis, P. guineae, P. leptonychotis, P. marincola, P. peli, P. segetis, P. taeanensis*	10	72.33–91.58	76.68–89.45
***P. fluorescens G***		134	–	–
***P. asplenii SG***	*P. agarici, P. asplenii, P. batumici, P. fuscovaginae, **P. vanderleydeniana***	5	84.41–89.66	84.10–88.35
***P. chlororaphis SG***	*P. chlororaphis* subsp. *aurantiaca, P. chlororaphis* subsp. *aureofaciens, P. chlororaphis* subsp. *chlororaphis, P. chlororaphis* subsp. *piscium*	4	97.83–98.45	94.73–96.95
***P. corrugata SG***	***P. alvandae*** *, P. beijieensis, P. brassicacearum, P. corrugata, P. kilonensis, **P. marvdashtae**, P. mediterranea, **P. tehranensis**, P. thivervalensis, P. viciae, **P. zanjanensis**, **P. zarinae***	12	89.75–97.36	85.55–95.75
***P. fluorescens SG***	*P. allii, P. antartica, **P. asgharzadehiana**, P. aylmerense, **P. azadiae**, P. azotoformans, P. canadensis, P. carnis, P. cedrina* subsp. *cedrina, P. cedrina* subsp. *fulgida, P. costantinii, P. cremoris, P. cyclaminis, P. edaphica, P. extremaustralis, P. extremorientalis, P. fildesensis, P. fluorescens, P. grimontii, P. haemolytica, P. kairouanesis, P. karstica, **P. khavaziana**, P. kitaguniensis, P. lactis, P. libanensis, P. lurida, P. marginalis, P. nabeulensis, P. orientalis, P. palleroniana, P. panacis, P. paracarnis, P. paralactis, P. pisciculturae, P. poae, P. rhodesiae, **P. salmasensis**, P. salomonii, P. simiae, P. sivasensis, P. spelaei, P. synxantha, P. tolaasii, **P. tritici**, P. trivialis, P. veronii, P. yamanorum*	48	85.16–98.91	83.52–95.68
***P. fragi SG***	*P. bubulae, P. deceptionensis, P. endophytica, P. fragi, P. helleri, P. lundensis, P. psychrophila, P. saxonica, P. taetrolens, P. versuta, P. weihenstephanensis*	11	83.00–97.67	80.50–90.49
***P. gessardii SG***	*P. brennerii, P. gessardii, P. mucidolens, P. proteolytica, **P. shahriarae***	5	90.57–97.53	85.57–92.54
***P. jessenii SG***	***P. asgharzadehiana*** *, **P. azerbaijanoccidens**, P. izuensis, P. jessenii, P. laurylsulfatiphila, P. laurylsulfatovorans, P. mohnii, P. moorei, P. reinekei, P. umsongensis, P. vancouverensis*	11	90.37–100	84.91–95.51
***P. koreensis SG***	*P. atacamensis, P. atagosis, P. baetica, **P. bananamidigenes**, **P. botevensis**, P. crudilactis, **P. ekonensis**, P. glycinae, P. granadensis, **P. hamedanensis**, P. helmanticensis, **P. iranensis**, **P. khorasanensis**, P. koreensis, P. kribbensis, **P. monsensis**, P. moraviensis, P. neuropathica, **P. siliginis**, **P. tensinigenes**, **P. triticicola**, **P. zeae***	22	85.40–99.53	82.48–96.09
***P. mandelii SG***	*P. arsenicoxydans, **P. farris**, P. frederiksbergensis, P. gregormendelii, P. lini, P. mandelii, P. migulae, P. mucoides, P. piscium, P. prosekii, P. silesiensis*	11	91.04–96.89	84.68–94.29
***P. protegens SG***	*P. aestus, P. protegens, P. saponiphila, **P. sessilinigenes***	4	89.52–95.57	86.41–91.86
***P. kielensis SG***	*P. kielensis*	1	–	–
***P. linyingensis G***	*P. guangdongensis, P. linyingensis, P. oryzae, P. sagittaria*	4	79.94–93.85	85.19–92.01
***P. lutea G***	*P. abietaniphila, P. bohemica, P. graminis, P. lutea*	4	83.31–88.30	81.89–85.81
***P. oleovorans G***	*P. alcaliphila, P. chaetoceroseae, P. chengduensis, P. composti, P. guguanensis, P. hydrolytica, P. indoloxydans, P. khazarica, P. mendocina, P. oleovorans, P. pseudoalcaligenes, P. sediminis, P. sihuisensis, P. toyotomiensis*	14	88.51–98.76	86.06–95.79
***P. oryzihabitans G***	*P. asuensis, P. duriflava, P. luteola, P. oryzihabitans, P. psychrotolerans, P. rhizoryzae, P. zeshuii*	7	66.46–94.18	73.64–88.62
***P. pertucinogena G***	*P. abyssi, P. aestusnigri, P. bauzanensis, P. formosensis, P. gallaeciencis, P. jilinensis, P. litoralis, P. oceani, P. pachastrellae, P. pelagia, P. pertucinogena, P. phragmitis, P. populi, P. profundi, P. sabulinigri, P. salegens, P. salina, P. saliphila, P. saudimassiliensis, P. xiamenensis, P. xinjiangensis, P. yangmingensis*	22	64.53–92.98	74.65–89.65
***P. putida G***		51		
***P. akappagea SG***	*P. akappagea*	1	–	–
***P. japonica SG***	*P. brassicae, P. defluvii, P. huaxiensis, P. japonica, P. laurentiana, P. qingdaonensis*	6	82.69–95.05	80.96–91.58
***P. vranovensis SG***	*P. alkylphenolica, P. donghuensis, P. hutmensis, P. tructae, P. vranovensis, P. wadenswillerensis*	6	84.23–94.10	84.52–93.03
***P. cremoricolorata SG***	*P. cremoricolorata*	1	–	–
***P. reidholzensis SG***	*P. reidholzensis, **P. shirazensis**, **P. urmiensis***	3	85.78–92.89	84.27–86.77
***P. guariconensis SG***	*P. guariconensis*	1	–	–
***P. wayambapalatensis SG***	***P. wayambapalatensis***	1	–	–
***P. farsensis SG***	***P. farsensis***	1	–	–
***P. xanthosomae SG***	***P. fakonensis, P. xanthosomae***	2	97.84	95.06
***P. mosselii SG***	*P. entomophila, **P. maumuensis**, P. mosselii, **P. muyukensis**, **P. peradeniyensis**, P. sichuanensis, P. soli, **P. xantholysinigenes***	8	87.48–95.35	87.35–94.87
***P. taiwanensis SG***	*P. taiwanensis*	1	–	–
***P. plecoglossicida SG***	*P. plecoglossicida*	1	–	–
***P. vlassakiae SG***	*P. hunanensis, **P. promysalinigenes, P. vlassakiae***	3	89.34–93.04	86.58
***P. capeferrum SG***	*P. capeferrum, **P. kermanshahensis***	2	93.01	90.26
***P. putida SG***	*P. alloputida, **P. anuradhapurensis**, P. asiatica, P. fulva, P. inefficax, P. juntendi, **P. kurunegalensis**, P. monteilii, **P. oryzicola**, P. parafulva, P. putida, P. pyomelaminifaciens, P. persica, P. shirazica*	14	85.32–97.99	82.66–95.79
***P. resinovorans G***	*P. furukawaii, P. lalkuanensis, P. mangiferae, P. otitidis, P. resinovorans*	5	81.47–90.46	79.25–87.84
***P. straminea G***	*P. argentinensis, P. daroniae, P. dryadis, P. flavescens, P. punonensis, P. seleniipraecipitans, P. straminea*	7	86.02–93.63	82.83–88.54
***P. stutzeri G***	*P. azotofigens, P. balearica, P. chloritidismutans, P. kirkiae, P. kunmingensis, P. nitritititolerans, P. nosocomialis, P. perfectomarina, P. saudiphocaensis, P. songnenensis, P. stutzeri, P. urumqiensis, P. xanthomarina, P. zhaodongensis*	14	73.60–89.69	76.39–88.15
***P. syringae G***	*P. amygdali, P. asturieensis, P. avellanae, P. cannabina, P. caricapapayae, P. caspiana, P. cerasi, P. cichorii, P. congelans, P. coronafaciens, P. ficuserectae, P. floridensis, P. meliae, P. ovata, P. savastanoi, P. syringae, P. tremae, P. viridiflava*	18	78.36–99.54	78.20–94.57
**Newly described groups**				
***P. pohangensis G***	*P. mangrovi, P. pohangensis*	2	67.02	77.07
***P. massiliensis G***	*P. massiliensis, P. typographi*	2	80.53	76.86
***P. rhizosphaerae G***	*P. baltica, P. coleopterorum, P. rhizosphaerae*	3	91.89–94.70	88.28–90.55
**Orphan groups**				
***P. indica G***	*P. indica*	1	–	–
***P. kuykendallii G***	*P. kuykendallii*	1	–	–
***P. thermotolerans G***	*P. thermotolerans*	1	–	–
***P. flexibilis G***	*P. flexibilis, P. tuomuerensis*	2	–	–
***P. fluvialis G***	*P. fluvialis, P. pharmacofabricae*	2	–	–
***P. alcaligenes G***	*P. alcaligenes*	1	–	–
***P. matsuisoli G***	*P. matsuisoli*	1	–	–
***P. turukhanskensis G***	*P. turukhanskensis*	1	–	–

^1^ Excluding synonymous species (Table 2).

**Table 2:** Synonymous species of *Pseudomonas* (Table S7). Species are considered synonymous when ANIb values are greater than or equal to 96.5% [18].

Groups/Subgroups	*Pseudomonas* Species		ANIb	Earlier Synonyms
***P. aeruginosa* group**	*P. citronellolis*	*P. humi*	96.70	*P. citronellolis*
	*P. nitroreducens*	*P. nitritireducens*	98.85	*P. nitroreducens*
***P. oleovorans* group**	*P. oleovorans*	*P. pseudoalcaligenes*	97.17	*P. oleovorans*
	*P. chengduensis*	*P. sihuiensis*	96.25	*P. chengduensis*
***P. oryzihabitans* group**	*P. oryzihabitans*	*P. psychrotolerans*	98.22	*P. oryzihabitans*
	*P. luteola*	*P. zeshuii*	97.87	*P. luteola*
***P. pertucinogena* group**	*P. phragmitis*	*P. jilinensis*	98.70	*P. phragmitis*
	*P. gallaeciensis*	*P. abyssi*	97.56	*P. gallaeciensis*
***P. putida* group**	*P. asiatica*	*P. pyomelaninifaciens*	99.03	*P. asiatica*
		*P. shirazica*	99.17	
***P. stutzeri* group**	*P. chloritidismutans*	*P. kunmingensis*	96.49	*P. chloritidismutans*
***P. syringae* group**	*P. tremae*	*P. coronafaciens*	98.74	*P. tremae*
	*P. amygdali*	*P. ficuserectae*	97.42	*P. amygdali*
		*P. meliae*	98.27	
		*P. savastanoi*	98.75	
***P. fluorescens* group**	*P. asplenii*	*P. fuscovaginae*	98.23	*P. asplenii*
	*P. veronii*	*P. panacis*	99.95	*P. veronii*
**Orphan groups**	*P. flexibilis*	*P. tuomuerensis*	98.69	*P. flexibilis*
	*P. fluvialis*	*P. pharmacofabricae*	98.61	*P. fluvialis*

**Table 3:** Phylogenetic affiliation based on ANIb values for the 44 whole genome sequenced strains belonging to the *P. putida* group, previously not assigned, or incorrectly assigned at the species level. Accession numbers are shown in Table S6.

Subgroups	Strain	Closest Type Strain	ANIb %	Re-identified Species
*P. japonica*	*P. putida* CSV86	*P. japonica*	86.94	*Pseudomonas* sp. #1
*P. reidholzensis*	*P. putida* 02C-26	*P. shirazensis*	97.25	*P. shirazensis*
*P. guariconensis*	*P. putida* IEC33019	*P. guariconensis*	91.47	*Pseudomonas* sp. #2
	*P. putida* WP4-W18-CRE-03	*P. guariconensis*	99.38	*P. guariconensis*
	*P. putida* WP8-W18-CRE-01		99.47	
*P. wayambapalatensis*	*P. putida* NX-1	*P. wayambapalatensis*	94.69	*Pseudomonas* sp. #3
	*P. putida* PC2		94.78	
	*Pseudomonas* sp. RW3S2	*P. wayambapalatensis*	99.21	*P. wayambapalatensis*
	*Pseudomonas* sp. RW10S2		99.26	
*P. farsensis*	*Pseudomonas* sp. SWRI51	*P. farsensis*	98.65	*P. farsensis*
*P. mosselii*	*Pseudomonas* sp. 250J	*P. peradeniyensis*	96.15*	*Pseudomonas* sp. #4
	*Pseudomonas* sp. BW16M2	*P. peradeniyensis*	96.59	*P. peradeniyensis*
*P. plecoglossicida*	*P. putida* GM84	*P. plecoglossicida*	91.13	*Pseudomonas* sp. #5
	*P. putida* GIMC5401-PPKH-115	*P. plecoglossicida*	87.01	*Pseudomonas* sp. #6
	*P. putida* BR-PH17	*P. plecoglossicida*	86.75	*Pseudomonas* sp. #7
	*P. putida* W619		85.75	
*P. vlassakiae*	*P. putida* AA7	*P. vlassakiae*	90.88	*Pseudomonas* sp. #8
	*P. putida* W5	*P. vlassakiae*	91.64	*Pseudomonas* sp. #9
*P. capeferrum*	*Pseudomonas* sp. SWRI68	*P. capeferrum*	98.66	*P. capeferrum*
	*Pseudomonas* sp. SWRI59		98.65	
	*P. putida* E41	*P. kermanshahensis*	97.48	*P. kermanshahensis*
	*P. putida* E46		97.55	
	*Pseudomonas* sp. SWRI50		99.39	
	*Pseudomonas* sp. SWRI67		99.99	
*P. putida*	*P. putida* SY153	*P. jutendi*	98.15	*P. jutendi*
	*P. putida* TIJ-51		97.77	
	*P. putida* GB-1	*P. alloputida*	90.49	*Pseudomonas* sp. #10
	*P. putida* PP112420		90.54	
	*P. putida* S13-1-2	*P. putida*	94.55	*Pseudomonas* sp. #11
	*P. putida* KF715		93.73	*Pseudomonas* sp. #12
	*P. putida* ZXPA-20		93.40	
	*P. putida* H8234		93.33	
	*P. putida* B1		93.40	
	*P. putida* R51	*P. alloputida*	95.00*	*Pseudomonas* sp. #13
	*P. putida* BS3701	*P. alloputida*	96.67	*P. alloputida*
	*P. putida* MX-2		96.49	
	*P. putida* LS46		96.44	
	*P. putida* 15420352		96.42	
	*P. putida* YC-AE1		96.40	
	*P. putida* T25-27		96.51	
	*P. monteilii* 170620603RE	*P. kurunegalensis*	99.45	*P. kurunegalensis*
	*P. monteilii* 170918607		99.44	
	*P. monteilii* STW0522-72		99.64	
	*P. monteilii* FDAARGOS171		99.77	

* dDDH < 70%.

## Data Availability

Data is contained within the article or Supplementary Materials.

## Supplementary Materials

The following are available online at https://www.mdpi.com/article/10.3390/microorganisms9081766/s1, Figure S1: Genome-based phylogeny of the Pseudomonadaceae. Figure S2: Phylogenetic tree based on concatenated NRPS proteins from the Xantholysin family. Figure S3: Phylogenetic tree based on concatenated NRPS proteins from the Putisolvin family. Figure S4: Phylogenetic tree based on concatenated NRPS proteins of WLIP producers from the Viscosin family. Figure S5: Genome-based phylogeny of the *P. putida* group. Table S1: List of type strains used in this study. Table S2: List of environmental *Pseudomonas* isolates used to describe 43 new *Pseudomonas* species. Table S3: Phenotypic profiles of the 43 new *Pseudomonas* species. Table S4: *rpoD*-based affiliation of strains to newly described *Pseudomonas* species. Table S5: Whole genome-based affiliation of strains to newly described *Pseudomonas* species. Table S6: Prospection of CLP biosynthesis gene clusters within the *P. putida* group. Table S7: ANIb matrix including 313 type and 33 non-type strains of *Pseudomonas*. Table S8: *rpoD* similarity matrix based on 316 type strains of *Pseudomonas*.

## Appendix A. Descriptions of the 43 New Pseudomonas Species

The phenotypic descriptions are presented in Table S3. *RpoD* and whole genome-based assignment of additional *Pseudomonas* strains to the newly described species are shown, respectively, in Tables S1 and S2.

(#1) Description of *Pseudomonas anuradhapurensis* sp. nov.

*Pseudomonas anuradhapurensis* (a.nu.ra.dha.pur.en’sis. n.L. fem. adj. *anuradhapurensis*, from Anuradhapura, a city in Sri Lanka).

The type strain is RD8MR3^T^ (LMG 32021^T^ = CFBP 8837^T^) and was isolated from the endorhizosphere of rice, Anuradhapura, Sri Lanka in 1990. Its G + C content is 63.43 mol% (calculated based on its genome sequence). The 16S rRNA gene, *rpoD*, and whole-genome sequence of RD8MR3^T^ are publicly available through the accession numbers AM911640, MT621460, and CP077097, respectively.

(#2) Description of *Pseudomonas oryzicola* sp. nov.

*Pseudomonas oryzicola* (o.ry.zi’co.la. L. fem n. *Oryza* rice, L. suff. -cola (from L. n. *incola*) inhabitant dweller; N.L. n. *oryzicola*, rice dweller).

The type strain is RD9SR1^T^ (LMG 32022^T^ = CFBP 8838^T^) and was isolated from the exorhizosphere of rice, Anuradhapura, Sri Lanka in 1990. Its G + C content is 62.91 mol% (calculated based on its genome sequence). The 16S rRNA gene, *rpoD*, and whole-genome sequence of RD9SR1^T^ are publicly available through the accession numbers AM911646, MT621461, and JABWRZ000000000, respectively.

(#3) Description of *Pseudomonas kurunegalensis* sp. nov.

*Pseudomonas kurunegalensis* (ku.ru.ne.gal.en’sis. N.L. fem. adj. *kurunegalensis*, from Kurunegala, a city in Sri Lanka).

The type strain is RW1P2^T^ (LMG 32023^T^ = CFBP 8839^T^) and was isolated from the rhizoplane of rice, Kurunegala, Sri Lanka in 1990. Its G + C content is 62.09 mol% (calculated based on its genome sequence). The 16S rRNA gene, *rpoD*, and whole-genome sequence of RW1P2^T^ are publicly available through the accession numbers AM911650, MT621449, and JABWSB000000000, respectively.

(#4) Description of *Pseudomonas kermanshahensis* sp. nov.

*Pseudomonas kermanshahensis* (ker.man.shah.en’sis. N.L. fem. adj. *kermanshahensis*, from Kermanshah, a city in Iran).

The type strain is SWRI100^T^ (LMG 32035^T^ = CFBP 8840^T^) and was isolated from the rhizosphere of wheat (cultivar Marvdasht), Kermanshah, Iran in 2004. Its G + C content is 62.22 mol% (calculated based on its genome sequence). The *rpoD* and whole-genome sequence of SWRI100^T^ are publicly available through the accession numbers MT621423 and JABWRY000000000, respectively.

(#5) Description of *Pseudomonas wayambapalatensis* sp. nov.

*Pseudomonas wayambapalatensis* (wa.yam.ba.pa.lat.en’sis. N.L. fem. adj. *wayambapalatensis*, from Wayamba Palata, the name of the north-western province in Sri Lanka).

The type strain is RW3S1^T^ (LMG 32024^T^ = CFBP 8841^T^) and was isolated from the exorhizosphere of rice, Kurunegala, Sri Lanka in 1990. Its G + C content is 63.24 mol% (calculated based on its genome sequence). The 16S rRNA gene, *rpoD*, and whole-genome sequence of RW3S1^T^ are publicly available through the accession numbers AM911665, MT621434, and CP077096, respectively.

(#6) Description of *Pseudomonas xantholysinigenes* sp. nov.

*Pseudomonas xantholysinigenes* (xan.tho.ly.si.ni’ge.nes. N.L. neut. n. *xantholysinum*, xantholysin; Gr. v. *gennao* to produce; N.L. part. adj. *xantholysinigenes*, xantholysin producing).

The type strain is RW9S1A^T^ (LMG 32025^T^ = CFBP 8842^T^) and was isolated from the exorhizosphere of rice, Kurunegala, Sri Lanka in 1990. Its G + C content is 64.16 mol% (calculated based on its genome sequence). The 16S rRNA gene, *rpoD*, and whole-genome sequence of RW9S1A^T^ are publicly available through the accession numbers AM911667, MT621442, and CP077095, respectively.

(#7) Description of *Pseudomonas peradeniyensis* sp. nov.

*Pseudomonas peradeniyensis* (pe.ra.de.niy.en’sis. N.L. fem. adj. *peradeniyensis*, from Peradeniya, a city in Sri Lanka).

The type strain is BW13M1^T^ (LMG 32026^T^ = CFBP 8887^T^) and was isolated from banana plant endorhizosphere, Peradeniya, Sri Lanka in 1990. Its G + C content is 64.62 mol% (calculated based on its genome sequence). The *rpoD* and whole-genome sequence of BW13M1^T^ are publicly available through the accession numbers MT621446 and JABWRJ000000000, respectively.

(#8) Description of *Pseudomonas vlassakiae* sp. nov.

*Pseudomonas vlassakiae* (vlas.sak.i.a’e. N.L. gen. n. *vlassakiae*, from Katrien Vlassak, a Belgian microbiologist who isolated the strain RW4S2, in addition to RD3MR3, RD9SR1, RW1P2, RW3S1, RW9S1A, BW13M1, RW10S1, RW8P3, and BW11P2, which represent 10 new *Pseudomonas* species).

The type strain is RW4S2^T^ (LMG 32027^T^ = CFBP 8843^T^) and was isolated from the exorhizosphere of rice, Kurunegala, Sri Lanka in 1990. Its G + C content is 62.98 mol% (calculated based on its genome sequence). The 16S rRNA gene, *rpoD*, and whole-genome sequence of RW4S2^T^ are publicly available through the accession numbers AM911658, MT621428, and JABWRP000000000, respectively.

(#9) Description of *Pseudomonas promysalinigenes* sp. nov.

*Pseudomonas promysalinigenes* (pro.my.sa.li.ni’ge.nes. N.L. neut. n. *promysalinum*, promysalin; Gr. v. *gennao* to produce; N.L. part. adj. *promysalinigenes*, promysalin producing).

The type strain is RW10S1^T^ (LMG 32028^T^ = CFBP 8844^T^) and was isolated from the exorhizosphere of rice, Kurunegala, Sri Lanka in 1990. Its G + C content is 60.62 mol% (calculated based on its genome sequence). The 16S rRNA gene, *rpoD*, and whole-genome sequence of RW10S1^T^ are publicly available through the accession numbers AM911668, MT621430, and CP077094, respectively.

(#10) Description of *Pseudomonas urmiensis* sp. nov.

*Pseudomonas urmiensis* (ur.mi.en’sis. N.L. fem. adj. *urmiensis*, from Urmia, a city in Iran).

The type strain is SWRI10^T^ (LMG 32036^T^ = CFBP 8845^T^) and was isolated from the rhizosphere of wheat (cultivar Marvdasht), West Azerbaijan, Iran in 2004. Its G + C content is 61.81 mol% (calculated based on its genome sequence). The *rpoD* and whole-genome sequence of SWRI10^T^ are publicly available through the accession numbers MT621419 and JABWRE000000000, respectively.

(#11) Description of *Pseudomonas shirazensis* sp. nov.

*Pseudomonas shirazensis* (shi.raz.en’sis. N.L. fem. adj. *shirazensis*, from Shiraz, a city in Iran).

The type strain is SWRI56^T^ (LMG 32037^T^ = CFBP 8846^T^) and was isolated from the rhizosphere of wheat (cultivar Shiraz), Shiraz, Iran in 2004. Its G + C content is 61.85 mol% (calculated based on its genome sequence). The *rpoD* and whole-genome sequence of SWRI56^T^ are publicly available through the accession numbers MT621418 and JABWRD000000000, respectively.

(#12) Description of *Pseudomonas farsensis* sp. nov.

*Pseudomonas farsensis* (fars.en’sis. N.L. fem. adj. *farsensis*, from Fars, a province in Iran).

The type strain is SWRI107^T^ (LMG 32038^T^ = CFBP 8847^T^) and was isolated from the rhizosphere of wheat (cultivar Azadi), Shiraz, Iran in 2004. Its G + C content is 62.58 mol% (calculated based on its genome sequence). The *rpoD* and whole-genome sequence of SWRI107^T^ are publicly available through the accession numbers MT621411 and JABWRF000000000, respectively.

(#13) Description of *Pseudomonas vanderleydeniana* sp. nov.

*Pseudomonas vanderleydeniana* (van.der.ley.den.i.a’na. N.L. fem. adj. *vanderleydeniana*, from Jos Vanderleyden, a Belgian microbiologist who studied plant growth-promoting properties of root-associated alpha- and gammaproteobacteria, including nitrogen-fixing and fluorescent *Pseudomonas* isolates.

The type strain is RW8P3^T^ (LMG 32029^T^ = CFBP 8848^T^) and was isolated from the rhizoplane of rice, Kurunegala, Sri Lanka in 1990. Its G + C content is 62.97 mol% (calculated based on its genome sequence). The *rpoD* and whole-genome sequence of RW8P3^T^ are publicly available through the accession numbers MT621472 and CP077093, respectively.

(#14) Description of *Pseudomonas bananamidigenes* sp. nov.

*Pseudomonas bananamidigenes* (ba.na.na.mi.di’ge.nes. N.L. neut. n. *bananamidum*, bananamide; Gr. v. *gennao* to produce; N.L. part. adj. *bananamidigenes*, bananamide producing).

The type strain is BW11P2^T^ (LMG 32030^T^ = CFBP 8849^T^) and was isolated from banana plant exorhizosphere, Galagedara, Sri Lanka in 1990. Its G + C content is 60.62 mol% (calculated based on its genome sequence). The *rpoD* and whole-genome sequence of BW11P2^T^ are publicly available through the accession numbers MT621496 and LRUN00000000, respectively.

(#15) Description of *Pseudomonas iranensis* sp. nov.

*Pseudomonas iranensis* (i.ran.en’sis. N.L. fem. adj. *iranensis*, from Iran).

The type strain is SWRI54^T^ (LMG 32039^T^ = CFBP 8850^T^) and was isolated from the rhizosphere of wheat (cultivar Shiraz), Shiraz, Iran in 2004. Its G + C content is 59.89 mol% (calculated based on its genome sequence). The *rpoD* and whole-genome sequence of SWRI54^T^ are publicly available through the accession numbers MT621504 and CP077092, respectively.

(#16) Description of *Pseudomonas khorasanensis* sp. nov.

*Pseudomonas khorasanensis* (kho.ra.san.en’sis. N.L. fem. adj. *khorasanensis*, from Khorasan, a province in Iran).

The type strain is SWRI153^T^ (LMG 32040^T^ = CFBP 8851^T^) and was isolated from the rhizosphere of wheat (cultivar Kaasparoo), Khorasan, Iran in 2004. Its G + C content is 59.71 mol% (calculated based on its genome sequence). The *rpoD* and whole-genome sequence of SWRI153^T^ are publicly available through the accession numbers MT621508 and JABWQP000000000, respectively.

(#17) Description of *Pseudomonas hamedanensis* sp. nov.

*Pseudomonas hamedanensis* (ha.me.dan.en’sis. N.L. fem. adj. *hamedanensis*, from Hamedan, a city in Iran).

The type strain is SWRI65^T^ (LMG 32041^T^ = CFBP 8852^T^) and was isolated from the rhizosphere of wheat, Hamedan, Iran in 2004. Its G + C content is 59.99 mol% (calculated based on its genome sequence). The *rpoD* and whole-genome sequence of SWRI65^T^ are publicly available through the accession numbers MT621514 and CP077091, respectively.

(#18) Description of *Pseudomonas zeae* sp. nov.

*Pseudomonas zeae* (ze’ae. L. gen. n. *zeae*, from *Zea mays*, corn).

The type strain is OE 48.2^T^ (LMG 32031^T^ = CFBP 8853^T^) and was isolated from the rhizosphere of maize, in Belgium, ~1984–1985. Its G + C content is 58.99 mol% (calculated based on its genome sequence). The *rpoD* and whole-genome sequence of OE 48.2^T^ are publicly available through the accession numbers MT621498 and CP077090, respectively.

(#19) Description of *Pseudomonas tensinigenes* sp. nov.

*Pseudomonas tensinigenes* (ten.si.ni’ge.nes. N.L. neut. n. *tensinum*, tensin; Gr. v. *gennao* to produce; N.L. part. adj. *tensinigenes*, tensin producing).

The type strain is ZA 5.3^T^ (LMG 32032^T^ = CFBP 8882^T^) and was isolated from the rhizosphere of wheat, in Belgium, ~1984–1985. Its G + C content is 59.17 mol% (calculated based on its genome sequence). The *rpoD* and whole-genome sequence of ZA 5.3^T^ are publicly available through the accession numbers MT621501 and CP077089, respectively.

(#20) Description of *Pseudomonas monsensis* sp. nov.

*Pseudomonas monsensis* (mons.en’sis. N.L. fem. adj. *monsensis*, from Mons, a city in Belgium).

The type strain is PGSB 8459^T^ (LMG 32033^T^ = CFBP 8854^T^) and was isolated from the rhizosphere of maize, Mons, Belgium, ~1984–1985. Its G + C content is 60.05 mol% (calculated based on its genome sequence). The *rpoD* and whole-genome sequence of PGSB 8459^T^ are publicly available through the accession numbers MT621495 and CP077087, respectively.

(#21) Description of *Pseudomonas zanjanensis* sp. nov.

*Pseudomonas zanjanensis* (zan.jan.en’sis. N.L. fem. adj. *zanjanensis*, from Zanjan, a city in Iran).

The type strain is SWRI12^T^ (LMG 32042^T^ = CFBP 8855^T^) and was isolated from the rhizosphere of wheat (cultivar Alvand), Zanjan, Iran in 2004. Its G + C content is 61.21 mol% (calculated based on its genome sequence). The *rpoD* and whole-genome sequence of SWRI12^T^ are publicly available through the accession numbers MT621484 and JABWRB000000000, respectively.

(#22) Description of *Pseudomonas zarinae* sp. nov.

*Pseudomonas zarinae* (za.ri’nae. N.L. gen. n. *zarinae*, from Zarin, a wheat cultivar).

The type strain is SWRI108^T^ (LMG 32043^T^ = CFBP 8856^T^) and was isolated from the rhizosphere of wheat (cultivar Zarin), Kermanshah, Iran in 2004. Its G + C content is 60.86 mol% (calculated based on its genome sequence). The rpoD and whole-genome sequence of SWRI108^T^ are publicly available through the accession numbers MT621493 and CP077086, respectively.

(#23) Description of *Pseudomonas tehranensis* sp. nov.

*Pseudomonas tehranensis* (teh.ran.en’sis. N.L. fem. adj. *tehranensis*, from Tehran, a city in Iran).

The type strain is SWRI196^T^ (LMG 32044^T^ = CFBP 8857^T^) and was isolated from the rhizosphere of wheat, Tehran, Iran in 2004. Its G + C content is 60.46 mol% (calculated based on its genome sequence). The *rpoD* and whole-genome sequence of SWRI196^T^ are publicly available through the accession numbers MT621473 and JABWQV000000000, respectively.

(#24) Description of *Pseudomonas marvdashtae* sp. nov.

*Pseudomonas marvdashtae* (marv.dash’tae. N.L. gen. n. *marvdashtae*, from Marvdasht, a wheat cultivar).

The type strain is SWRI102^T^ (LMG 32045^T^ = CFBP 8858^T^) and was isolated from the rhizosphere of wheat (cultivar Marvdasht), Kermanshah, Iran in 2004. Its G + C content is 60.64 mol% (calculated based on its genome sequence). The *rpoD* and whole-genome sequence of SWRI102^T^ are publicly available through the accession numbers MT621490 and JABWQX000000000, respectively.

(#25) Description of *Pseudomonas shahriarae* sp. nov.

*Pseudomonas shahriarae* (shah.ri.a’rae. N.L. gen. n. *shahriarae*, from Shahriar, a wheat cultivar).

The type strain is SWRI52^T^ (LMG 32046^T^ = CFBP 8859^T^) and was isolated from the rhizosphere of wheat (cultivar Shahriar), Zanjan, Iran in 2004. Its G + C content is 60.59 mol% (calculated based on its genome sequence). The *rpoD* and whole-genome sequence of SWRI52^T^ are publicly available through the accession numbers MT621521 and CP077085, respectively.

(#26) Description of *Pseudomonas azadiae* sp. nov.

*Pseudomonas azadiae* (a.za’di.ae. N.L. gen. n. *azadiae*, from Azadi, a wheat cultivar).

The type strain is SWRI103^T^ (LMG 32047^T^ = CFBP 8860^T^) and was isolated from the rhizosphere of wheat (cultivar Azadi), Shiraz, Iran in 2004. Its G + C content is 60.69 mol% (calculated based on its genome sequence). The *rpoD* and whole-genome sequence of SWRI103^T^ are publicly available through the accession numbers MT621536 and JAHSTY000000000, respectively.

(#27) Description of *Pseudomonas tritici* sp. nov.

*Pseudomonas tritici* (tri’ti.ci. L. gen. n. tritici, of Triticum, wheat).

The type strain is SWRI145^T^ (LMG 32048^T^ = CFBP 8883^T^) and was isolated from the rhizosphere of wheat, Zanjan, Iran in 2004. Its G + C content is 59.87 mol% (calculated based on its genome sequence). The *rpoD* and whole-genome sequence of SWRI145^T^ are publicly available through the accession numbers MT621537 and CP077084, respectively.

(#28) Description of *Pseudomonas salmasensis* sp. nov.

*Pseudomonas salmasensis* (sal.mas.en’sis. N.L. fem. adj. *salmasensis*, from Salmas, a city in Iran).

The type strain is SWRI126^T^ (LMG 32049^T^ = CFBP 8861^T^) and was isolated from the rhizosphere of wheat (cultivar Zarin), Salmas, Iran in 2004. Its G + C content is 60.16 mol% (calculated based on its genome sequence). The *rpoD* and whole-genome sequence of SWRI126^T^ are publicly available through the accession numbers MT621526 and CP077083, respectively.

(#29) SWRI22 (LMG 32050 = CFBP 8862): *Pseudomonas carnis*

(#30) OE 28.3 (LMG 32034 = CFBP 8863): *Pseudomonas edaphica*

(#31) SWRI76 (LMG 32051 = CFBP 8864): *Pseudomonas atacamensis*

(#32) Description of *Pseudomonas triticicola* sp. nov.

*Pseudomonas triticicola* (tri.ti.ci.co’la. L. fem. n. *Triticum*, wheat; L. suff. –cola (from L. n. *incola*), inhabitant dweller; N.L. n. *triticicola*, wheat dweller).

The type strain is SWRI88^T^ (LMG 32052^T^ = CFBP 8865^T^) and was isolated from the rhizosphere of wheat (cultivar Marvdasht), Kermanshah, Iran in 2004. Its G + C content is 59.99 mol% (calculated based on its genome sequence). The whole-genome sequence of SWRI88^T^ is publicly available through the accession JAHSTX000000000.

(#33) Description of *Pseudomonas siliginis* sp. nov.

*Pseudomonas siliginis* (si.li’gi.nis. L. gen. n. *siliginis*, of siligo, winter wheat).

The type strain is SWRI31^T^ (LMG 32053^T^ = CFBP 8866^T^) and was isolated from the rhizosphere of wheat (cultivar Zarin), Kermanshah, Iran in 2004. Its G + C content is 59.97 mol% (calculated based on its genome sequence). The whole-genome sequence of SWRI31^T^ is publicly available through the accession JAHSTW000000000.

(#34) Description of *Pseudomonas farris* sp. nov.

*Pseudomonas farris* (far’ris. L. gen. n. *farris*, of husked wheat, of a grain).

The type strain is SWRI79^T^ (LMG 32054^T^ = CFBP 8867^T^) and was isolated from the rhizosphere of wheat (cultivar Local), Zanjan, Iran in 2004. Its G + C content is 58.74 mol% (calculated based on its genome sequence). The whole-genome sequence of SWRI79^T^ is publicly available through the accession JAHSTV000000000.

(#35) Description of *Pseudomonas azerbaijanoccidens* sp. nov.

*Pseudomonas azerbaijanoccidens* (a.zer.bai.jan.oc’ci.dens. Azerbaijan, geographic name; L. fem. adj. *occidens*, western; N.L. fem. adj. *azerbaijanoccidens*, from West Azerbaijan, a province in Iran).

The type strain is SWRI74^T^ (LMG 32055^T^ = CFBP 8868^T^) and was isolated from the rhizosphere of wheat (cultivar Zarin), Salmas, Iran in 2004. Its G + C content is 59.30 mol% (calculated based on its genome sequence). The whole-genome sequence of SWRI74^T^ is publicly available through the accession JAHSTU000000000.

(#36) Description of *Pseudomonas alvandae* sp. nov.

*Pseudomonas alvandae* (al.van’dae. N.L. gen. n. *alvandae*, from Alvand, a wheat cultivar).

The type strain is SWRI17^T^ (LMG 32056^T^ = CFBP 8869^T^) and was isolated from the rhizosphere of wheat (cultivar Alvand), Zanjan, Iran in 2004. Its G + C content is 60.86 mol% (calculated based on its genome sequence). The whole-genome sequence of SWRI17^T^ is publicly available through the accession CP077080.

(#37) Description of *Pseudomonas asgharzadehiana* sp. nov.

*Pseudomonas asgharzadehiana* (as.ghar.za.deh.i.a’na. N.L. fem. adj. *asgharzadehiana*, from Ahmad Asgharzadeh, an Iranian microbiologist who, together with Kazem Khavazi, isolated the strain SWRI132, in addition to SWRI10, SWRI12, SWRI17, SWRI31, SWRI52, SWRI54, SWRI56, SWRI65, SWRI74, SWRI79, SWRI88, SWRI100, SWRI102, SWRI103, SWRI107, SWRI108, SWRI123, SWRI124, SWRI126, SWRI145, SWRI153, and SWRI196, which represent 23 new *Pseudomonas* species).

The type strain is SWRI132^T^ (LMG 32057^T^ = CFBP 8870^T^) and was isolated from the rhizosphere of wheat (cultivar Marvdasht), Kermanshah, Iran in 2004. Its G + C content is 60.59 mol% (calculated based on its genome sequence). The whole-genome sequence of SWRI132^T^ is publicly available through the accession CP077079.

(#38) Description of *Pseudomonas azerbaijanoriens* sp. nov.

*Pseudomonas azerbaijanoriens* (a.zer.bai.jan.o’ri.ens. Azerbaijan, geographic name; L. fem. adj. *oriens*, eastern; N.L. fem. adj. *azerbaijanoriens*, from East Azerbaijan, a province in Iran).

The type strain is SWRI123^T^ (LMG 32058^T^ = CFBP 8871^T^) and was isolated from the rhizosphere of wheat (cultivar Zarin), East Azerbaijan, Iran in 2004. Its G + C content is 60.11 mol% (calculated based on its genome sequence). The whole-genome sequence of SWRI123^T^ is publicly available through the accession CP077078.

(#39) Description of *Pseudomonas khavaziana* sp. nov.

*Pseudomonas khavaziana* (kha.va.zi.a’na. N.L. fem. adj. *khavaziana*, from Kazem Khavazi, an Iranian microbiologist who, together with Ahmad Asgharzadeh, isolated the strain SWRI124, in addition to SWRI10, SWRI12, SWRI17, SWRI31, SWRI52, SWRI54, SWRI56, SWRI65, SWRI74, SWRI79, SWRI88, SWRI100, SWRI102, SWRI103, SWRI107, SWRI108, SWRI123, SWRI126, SWRI132, SWRI145, SWRI153, and SWRI196, which represent 23 new *Pseudomonas* species).

The type strain is SWRI124^T^ (LMG 32059^T^ = CFBP 8872^T^) and was isolated from the rhizosphere of wheat (cultivar Zarin), East Azerbaijan, Iran in 2004. Its G + C content is 59.61 mol% (calculated based on its genome sequence). The whole-genome sequence of SWRI124^T^ is publicly available through the accession JAHSTT000000000.

(#40) Description of *Pseudomonas botevensis* sp. nov.

*Pseudomonas botevensis* (bo.tev.en’sis. N.L. fem. adj. *botevensis*, from Boteva, a city in Cameroon).

The type strain is COW3^T^ (LMG 32176^T^ = CFBP 8873^T^) and was isolated from the roots of white cocoyam (*Xanthosoma sagittifolium*), Boteva, Cameroon in 2008. Its G + C content is 61.21 mol% (calculated based on its genome sequence). The 16S rRNA gene, *rpoB*, *gyrB*, *rpoD*, and whole-genome sequence of COW3^T^ are publicly available through the accession numbers MT507065, MT506178, MT506955, MT506158, and JAHTKI000000000 respectively.

(#41) Description of *Pseudomonas ekonensis* sp. nov.

*Pseudomonas ekonensis* (e.kon.en’sis. N.L. fem. adj. *ekonensis*, from Ekona, a city in Cameroon).

The type strain is COR58^T^ (LMG 32175^T^ = CFBP 8874^T^) and was isolated from the roots of red cocoyam (*Xanthosoma sagittifolium*), Ekona, Cameroon in 2008. Its G + C content is 64.87 mol% (calculated based on its genome sequence). The 16S rRNA gene, *rpoB*, *gyrB*, *rpoD*, and whole-genome sequence of COR58^T^ are publicly available through the accession numbers MT507072, MT506185, MT506962, MT506165, and JAHSTS000000000, respectively.

(#42) Description of *Pseudomonas maumuensis* sp. nov.

*Pseudomonas maumuensis* (mau.mu.en’sis. N.L. fem. adj. *maumuensis*, from Maumu, a city in Cameroon).

The type strain is COW77^T^ (LMG 32179^T^ = CFBP 8888^T^) and was isolated from the roots of white cocoyam (*Xanthosoma sagittifolium*), Maumu, Cameroon in 2008. Its G + C content is 64.12 mol% (calculated based on its genome sequence). The *rpoB*, *rpoD*, and whole-genome sequence of COW77^T^ are publicly available through the accession numbers MH594167, MK251918, and CP077077, respectively.

(#43) Description of *Pseudomonas fakonensis* sp. nov.

*Pseudomonas fakonensis* (fa.kon.en’sis. N.L. fem. adj. *fakonensis*, from Fako, a county in Cameroon).

The type strain is COW40^T^ (LMG 32178^T^ = CFBP 8875^T^) and was isolated from the roots of white cocoyam (*Xanthosoma sagittifolium*), Ekona (Fako county), Cameroon in 2008. Its G + C content is 64.27 mol% (calculated based on its genome sequence). The *rpoB*, *rpoD*, and whole-genome sequence of COW40^T^ are publicly available through the accession numbers MH594146, MK251899, and CP077076, respectively.

(#44) Description of *Pseudomonas xanthosomae* sp. nov.

*Pseudomonas xanthosomae* (xan.tho.so’mae. L. gen. n. *xanthosomae*, of *Xanthosoma*, cocoyam).

The type strain is COR54^T^ (LMG 32174^T^ = CFBP 8876^T^) and was isolated from the roots of red cocoyam (*Xanthosoma sagittifolium*), Ekona, Cameroon in 2008. Its G + C content is 64.19 mol% (calculated based on its genome sequence). The *rpoB*, *rpoD*, and whole-genome sequence of COR54^T^ are publicly available through the accession numbers MH594196, MK251872, and CP077075, respectively.

(#45) CMR5c (LMG 32172 = CFBP 8889): *Pseudomonas aestus*

(#46) Description of *Pseudomonas sessilinigenes* sp. nov.

*Pseudomonas sessilinigenes* (ses.si.li.ni’ge.nes. N.L. neut. n. *sessilinum*, sessilin; Gr. v. *gennao* to produce; N.L. part. adj. *sessilinigenes*, sessilin producing).

The type strain is CMR12a^T^ (LMG 32173^T^ = CFBP 8877^T^) and was isolated from the roots of red cocoyam (*Xanthosoma sagittifolium*), Bokwai, Cameroon in 2001. Its G + C content is 62.80 mol% (calculated based on its genome sequence). The 16S rRNA gene, *rpoB*, *gyrB*, and whole-genome sequence of CMR12a^T^ are publicly available through the accession numbers FJ652622, FJ652703, FJ652730, and CP077074, respectively.

(#47) Description of *Pseudomonas muyukensis* sp. nov.

*Pseudomonas muyukensis* (mu.yuk.en’sis. N.L. fem. adj. *muyukensis*, from Muyuka, a district in Cameroon).

The type strain is COW39^T^ (LMG 32177^T^ = CFBP 8890^T^) and was isolated from the roots of white cocoyam (*Xanthosoma sagittifolium*), Ekona, Cameroon in 2008. Its G + C content is 65.11 mol% (calculated based on its genome sequence). The *rpoD* and whole-genome sequence of COW39^T^ are publicly available through the accession numbers MK329212 and CP077073, respectively.
