# Supplementary material for: The Ever-Expanding Pseudomonas Genus: Description of 43 New Species and Partition of the Pseudomonas putida Group
Source: Microorganisms. 2021 Aug 18;9(8):1766. doi: 10.3390/microorganisms9081766 (PMC8401041; doi:10.3390/microorganisms9081766)
Supplement: Supplementary file 1 [file microorganisms-09-01766-s001.zip › SupMatFigures_10-08-2021.pdf]

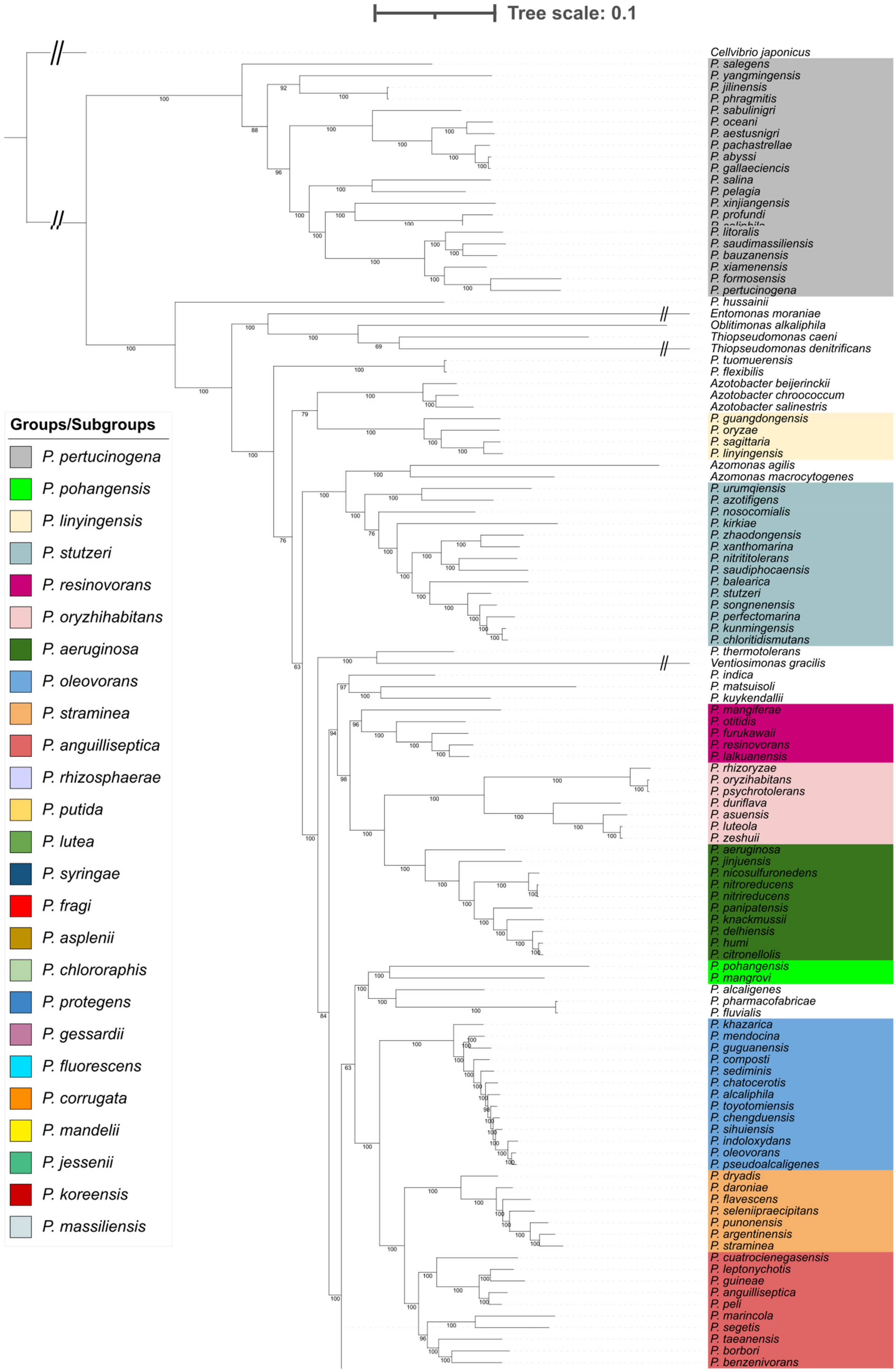

Tree scale: 0.1
Cellvibrio japonicus
P. salegens
P. yangmingensis
P. jilinensis
P. phragmitis
P. sabulinigri
P. oceani
P. aestusnigri
P. pachastrellae
P. abyssi
P. gallaeciensis
P. salina
P. pelagia
P. xinjiangensis
P. profundi
P. litoralis
P. saudimassiliensis
P. bauzanensis
P. xiamenensis
P. formosensis
P. pertucinogena
P. hussainii
Entomonas moraniae
Oblitimonas alkaliphila
Thiopseudomonas caeni
Thiopseudomonas denitrificans
P. tuomuerensis
P. flexibilis
Azotobacter beijerinckii
Azotobacter chroococcum
Azotobacter salinestris
P. guangdongensis
P. oryzae
P. sagittaria
P. linyingensis
Azomonas agilis
Azomonas macrocytogenes
P. urumqiensis
P. azotifigens
P. nosocomialis
P. kirkiae
P. zhaodongensis
P. xanthomarina
P. nitritireducens
P. saudiphocaensis
P. balearica
P. stutzeri
P. songnenensis
P. perfectomarina
P. kunmingensis
P. chloritidismutans
P. thermotolerans
Ventiosimonas gracilis
P. indica
P. matsuisoli
P. kuykendallii
P. mangiferae
P. otitidis
P. furukawaii
P. resinovorans
P. lalkuanensis
P. rhizoryzae
P. oryzihabitans
P. psychrotolerans
P. duriflava
P. asuensis
P. luteola
P. zeshuii
P. aeruginosa
P. jinjuensis
P. nicosulfuronedens
P. nitroreducens
P. nitrireducens
P. panipatensis
P. knackmussii
P. delhiensis
P. humi
P. citronellolis
P. pohangensis
P. mangrovi
P. alcaligenes
P. pharmacofabricae
P. fluvialis
P. khazarica
P. mendocina
P. guguanensis
P. composti
P. sediminis
P. chatocerotis
P. alcaliphila
P. toyotomiensis
P. chengduensis
P. sihuiensis
P. indoloxydans
P. oleovorans
P. pseudoalcaligenes
P. dryadis
P. daroniae
P. flavescens
P. seleniipraecipitans
P. punonensis
P. argentinensis
P. straminea
P. cuatrocienegasensis
P. leptonychotis
P. guineae
P. anguilliseptica
P. peli
P. marincola
P. segetis
P. taeanensis
P. borbori
P. benzenivorans
Groups/Subgroups
P. pertucinogena
P. pohangensis
P. linyingensis
P. stutzeri
P. resinovorans
P. oryzhihabitans
P. aeruginosa
P. oleovorans
P. straminea
P. anguilliseptica
P. rhizosphaerae
P. putida
P. lutea
P. syringae
P. fragi
P. asplenii
P. chlororaphis
P. protegens
P. gessardii
P. fluorescens
P. corrugata
P. mandelii
P. jessenii
P. koreensis
P. massiliensis

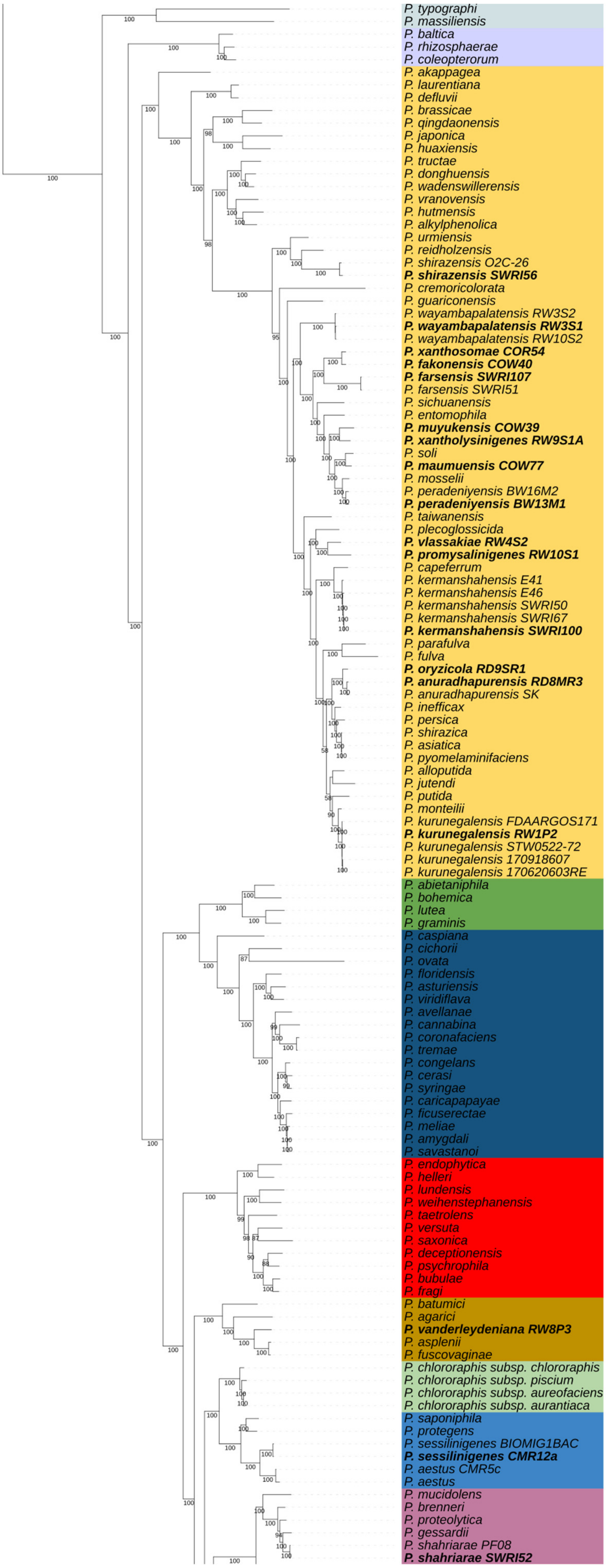
P. typographi
P. massiliensis
P. baltica
P. rhizosphaerae
P. coleopterorum
P. akappagea
P. laurentiana
P. defluvii
P. brassicae
P. qingdaonensis
P. japonica
P. huaxiensis
P. tructae
P. donghuensis
P. wadenswillerensis
P. vranovensis
P. hutmensis
P. alkylphenolica
P. urmiensis
P. reidholzensis
P. shirazensis O2C-26
P. shirazensis SWRI56
P. cremoricolorata
P. guariconensis
P. wayambapalatensis RW3S2
P. wayambapalatensis RW3S1
P. wayambapalatensis RW10S2
P. xanthosomae COR54
P. fakonensis COW40
P. farsensis SWRI107
P. farsensis SWRI51
P. sichuanensis
P. entomophila
P. muyukensis COW39
P. xantholysinigenes RW9S1A
P. soli
P. maumuensis COW77
P. mosselii
P. peradeniyensis BW16M2
P. peradeniyensis BW13M1
P. taiwanensis
P. plecoglossicida
P. vlassakiae RW4S2
P. promysalinigenes RW10S1
P. capeferrum
P. kermanshahensis E41
P. kermanshahensis E46
P. kermanshahensis SWRI50
P. kermanshahensis SWRI67
P. kermanshahensis SWRI100
P. parafulva
P. fulva
P. oryzicola RD9SR1
P. anuradhapurensis RD8MR3
P. anuradhapurensis SK
P. inefficax
P. persica
P. shirazica
P. asiatica
P. pyomelaminifaciens
P. alloputida
P. jutendi
P. putida
P. monteilii
P. kurunegalensis FDAARGOS171
P. kurunegalensis RW1P2
P. kurunegalensis STW0522-72
P. kurunegalensis 170918607
P. kurunegalensis 170620603RE
P. abietaniphila
P. bohemica
P. lutea
P. graminis
P. caspiana
P. cichorii
P. ovata
P. floridensis
P. asturiensis
P. viridiflava
P. avellanae
P. cannabina
P. coronafaciens
P. tremae
P. congelans
P. cerasi
P. syringae
P. caricapapayae
P. ficuserectae
P. meliae
P. amygdali
P. savastanoi
P. endophytica
P. helleri
P. lundensis
P. weihenstephanensis
P. taetrolens
P. versuta
P. saxonica
P. deceptionensis
P. psychrophila
P. bubulae
P. fragi
P. batumici
P. agarici
P. vanderleydeniana RW8P3
P. asplenii
P. fuscovaginae
P. chlororaphis subsp. chlororaphis
P. chlororaphis subsp. piscium
P. chlororaphis subsp. aureofaciens
P. chlororaphis subsp. aurantiaca
P. saponiphila
P. protegens
P. sessilinigenes BIOMIG1BAC
P. sessilinigenes CMR12a
P. aestus CMR5c
P. aestus
P. mucidolens
P. brenneri
P. proteolytica
P. gessardii
P. shahriarae PF08
P. shahriarae SWRI52

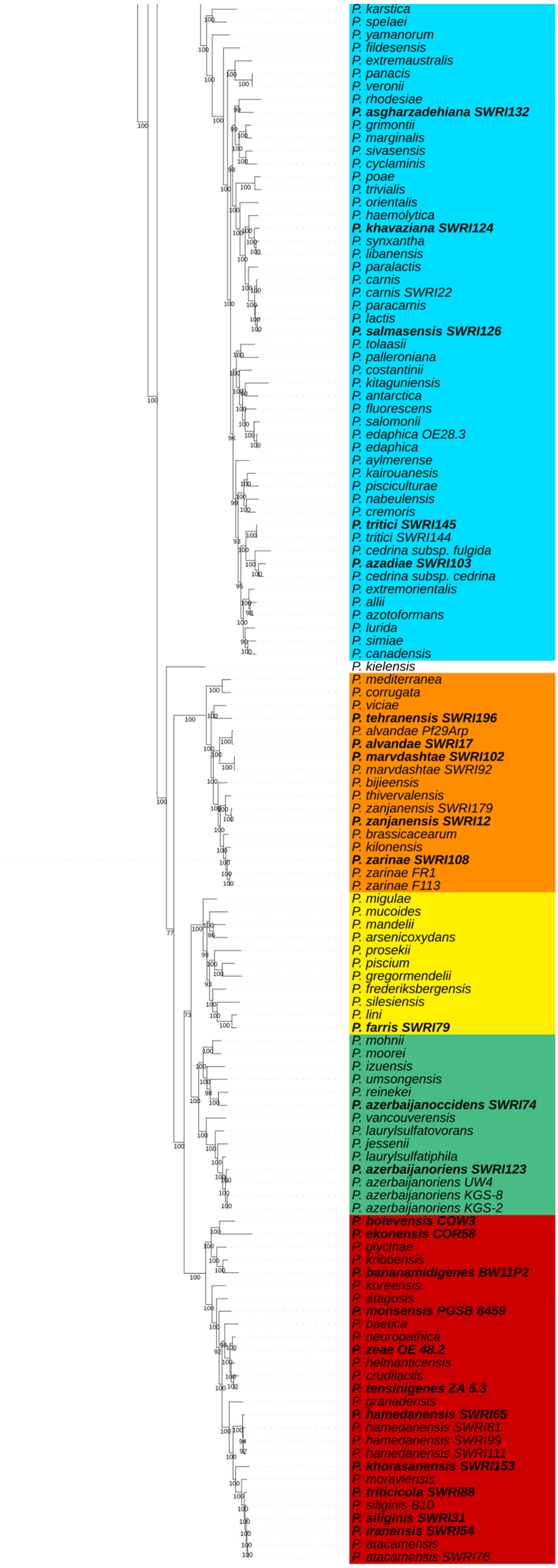
P. karstica
P. spelaei
P. yamanorum
P. fildesensis
P. extremaustralis
P. panacis
P. veronii
P. rhodesiae
P. asgharzadehiana SWRI132
P. grimontii
P. marginalis
P. sivasensis
P. cyclaminis
P. poae
P. trivialis
P. orientalis
P. haemolytica
P. khavaziana SWRI124
P. synxantha
P. libanensis
P. paralactis
P. carnis
P. carnis SWRI22
P. paracarnis
P. lactis
P. salmasensis SWRI126
P. tolaasii
P. palleroniana
P. costantinii
P. kitaguniensis
P. antarctica
P. fluorescens
P. salomonii
P. edaphica OE28.3
P. edaphica
P. aylmerense
P. kairouanensis
P. pisciculturae
P. nabeulensis
P. cremoris
P. tritici SWRI145
P. tritici SWRI144
P. cedrina subsp. fulgida
P. azadiae SWRI103
P. cedrina subsp. cedrina
P. extremorientalis
P. allii
P. azotoformans
P. lurida
P. simiae
P. canadensis
P. kielensis
P. mediterranea
P. corrugata
P. viciae
P. tehranensis SWRI196
P. alvandae Pf29Arp
P. alvandae SWRI17
P. marvdashtae SWRI102
P. marvdashtae SWRI92
P. bijieensis
P. thivervalensis
P. zanjanensis SWRI179
P. zanjanensis SWRI12
P. brassicacearum
P. kilonensis
P. zarinae SWRI108
P. zarinae FR1
P. zarinae F113
P. migulae
P. mucoides
P. mandelii
P. arsenicoxydans
P. prosekii
P. piscium
P. gregormendelii
P. frederiksbergensis
P. silesiensis
P. lini
P. farris SWRI79
P. mohnii
P. moorei
P. izuensis
P. umsongensis
P. reinekei
P. azerbaijanoccidens SWRI74
P. vancouverensis
P. laurylsulfatovorans
P. jessenii
P. laurylsulfatiphila
P. azerbaijanoriens SWRI123
P. azerbaijanoriens UW4
P. azerbaijanoriens KGS-8
P. azerbaijanoriens KGS-2
P. botevensis COW3
P. ekonensis COR58
P. glycinae
P. kribbensis
P. bananamidigenes BW11P2
P. koreensis
P. atagosis
P. monsensis PGSB 8459
P. baetica
P. neuropathica
P. zeae OE 48.2
P. helmanticensis
P. crudilactis
P. tensinigenes ZA 5.3
P. granadensis
P. hamedanensis SWRI65
P. hamedanensis SWRI81
P. hamedanensis SWRI99
P. hamedanensis SWRI111
P. khorasanensis SWRI153
P. moraviensis
P. triticicola SWRI88
P. siliginis B10
P. siliginis SWRI31
P. iranensis SWRI54
P. atacamensis
P. atacamensis SWRI76

Figure S1. Genome-based phylogeny of the *Pseudomonadaceae*. The phylogenetic tree was inferred using IQ-TREE with automatic model selection and 1,000 ultrafast bootstrap based on an alignment of 1,063 core orthogroup from the 272 *Pseudomonas* species (270 + *P. hussainii* and *P. caeni/Thiopseudomonas caeni*), the type strains of the newly described species and affiliated strains (n=76; Table S5) and type strains of other genera within the *Pseudomonadaceae* (n=9; Table S1). Newly described species are highlighted in bold. *Cellvibrio japonicus* is used as outgroup.

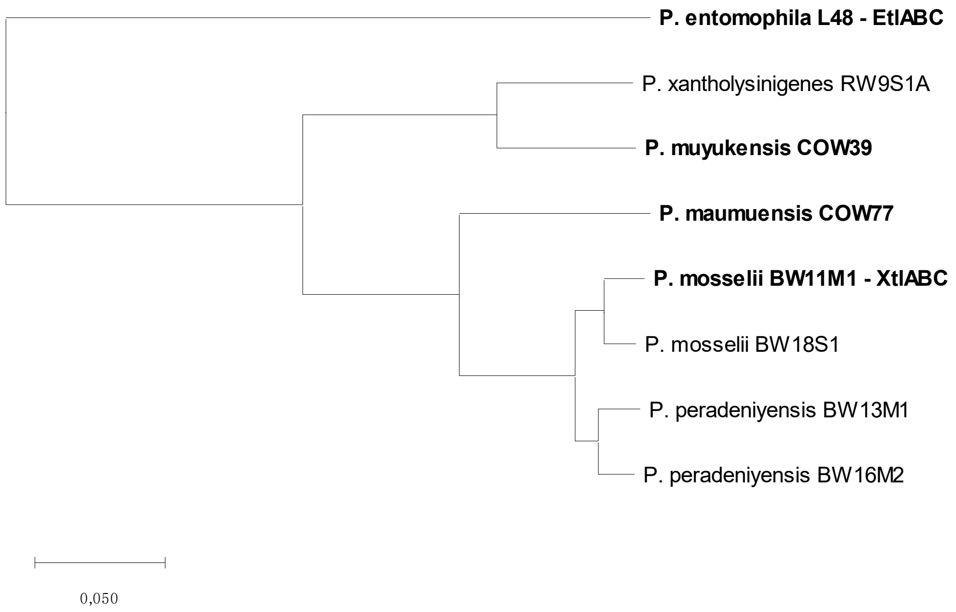

Figure S2. Phylogenetic tree based on concatenated NRPS proteins from the Xantholysin and Entolysin families. The maximum likelihood tree was constructed using the JTT+F+I+G model (MEGA-X). Characterized producers are highlighted in bold (Table S6). All Xantholysin and Entolysin producers belongs to the *P. mosselii* subgroup.

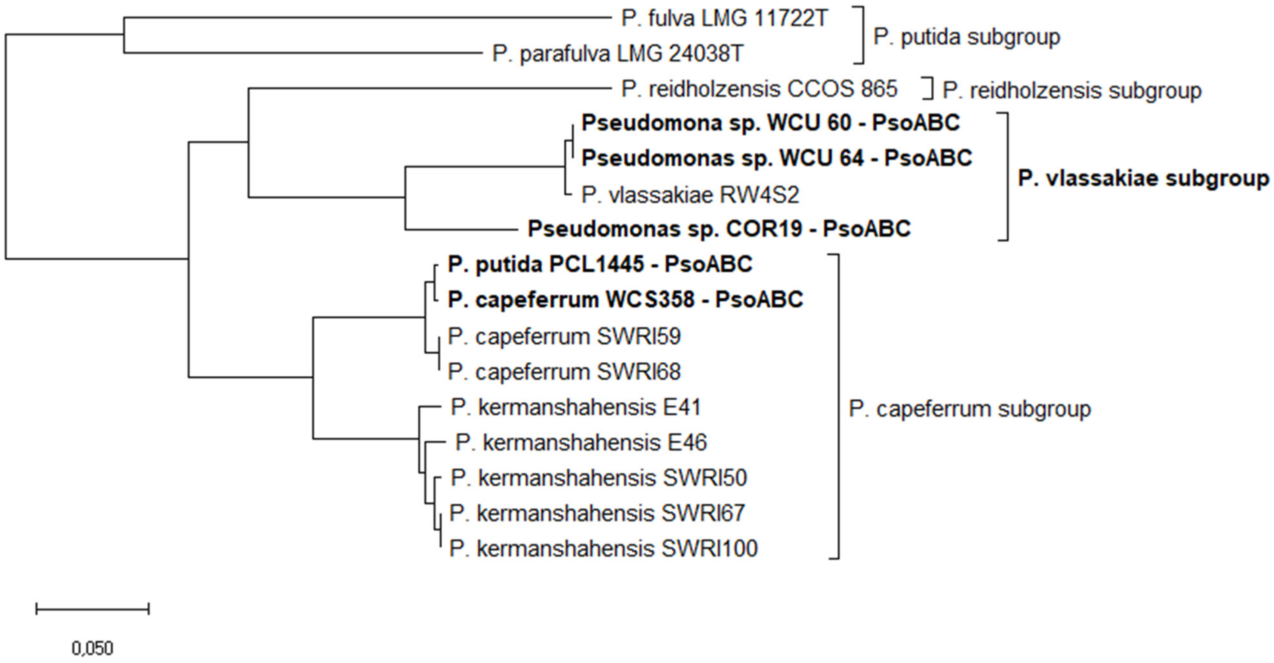

Figure S3. Phylogenetic tree based on concatenated NRPS proteins from the Putisolvin family. Maximum likelihood tree constructed using the JTT+F+I+G model (MEGA-X). Characterized producers are highlighted in bold (Table S6).

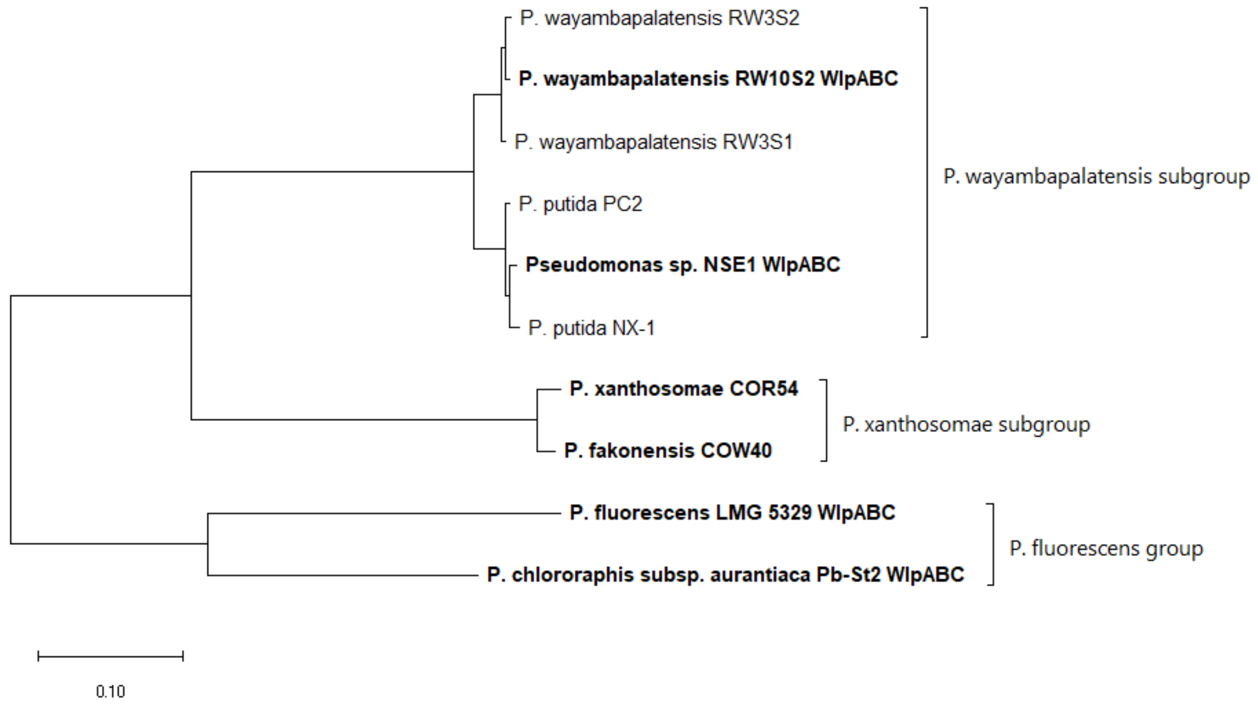

Figure S4. Phylogenetic tree based on concatenated NRPS proteins of WLIP producers from the Viscosin family. Maximum likelihood tree constructed using the JTT+F+I+G model (MEGA-X). Characterized producers are highlighted in bold (Table S6). NRPSs accession number for producers belonging to the *P. fluorescens* group: *P. fluorescens* LMG 5329 (WlpA AFZ61501; WlpB AFZ61525; WlpC AFZ61526) and *P. chlororaphis* subsp. *aurantiaca* PB-St2 (WlpA AZD30270; WlpB AZD30268; WlpC AZD30267).

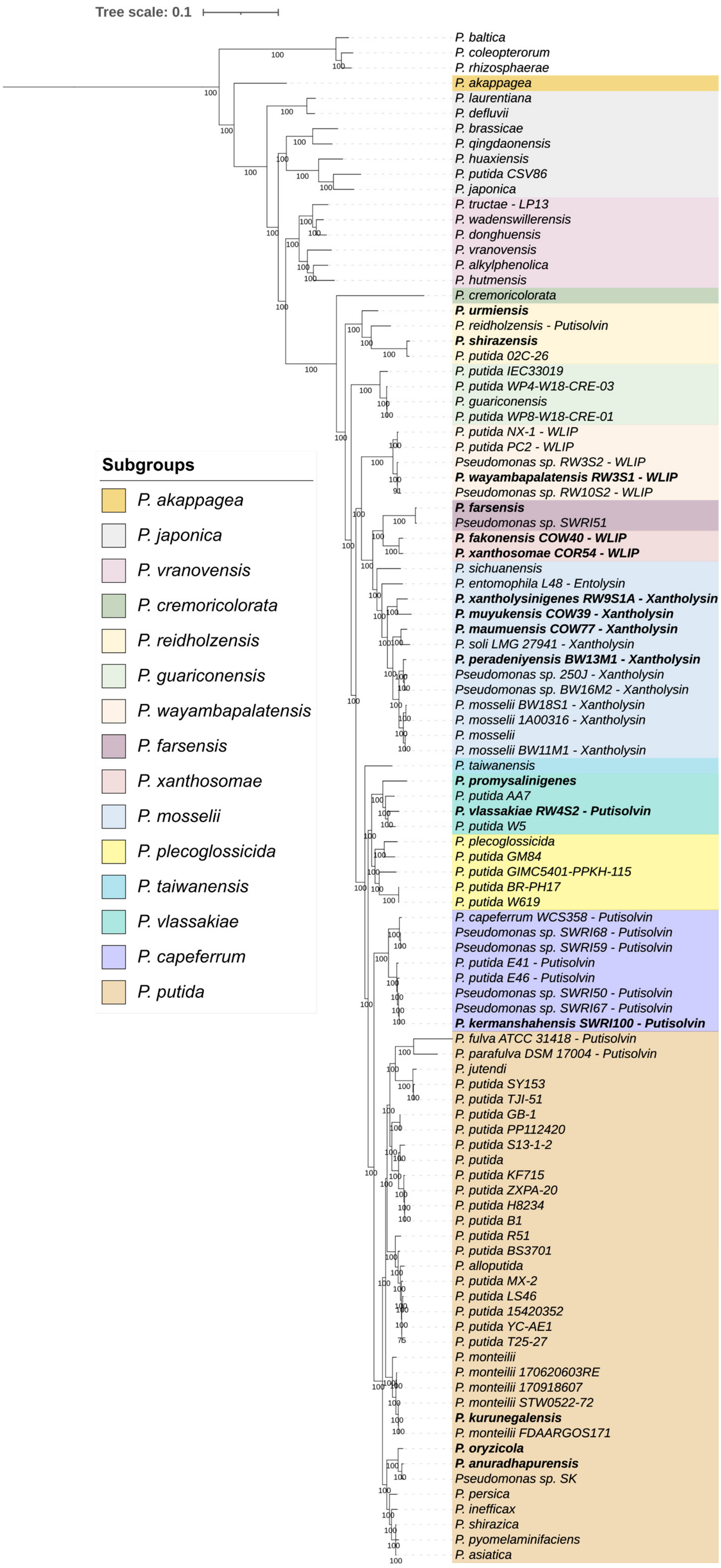
Tree scale: 0.1
P. baltica
P. coleopterorum
P. rhizosphaerae
P. akappagea
P. laurentiana
P. defluvii
P. brassicae
P. qingdaonensis
P. huaxiensis
P. putida CSV86
P. japonica
P. tructae - LP13
P. wadenswillerensis
P. donghuensis
P. vranovensis
P. alkylphenolica
P. hutmensis
P. cremoricolorata
P. urmiensis
P. reidholzensis - Putisolvin
P. shirazensis
P. putida 02C-26
P. putida IEC33019
P. putida WP4-W18-CRE-03
P. guariconensis
P. putida WP8-W18-CRE-01
P. putida NX-1 - WLIP
P. putida PC2 - WLIP
Pseudomonas sp. RW3S2 - WLIP
P. wayambapalatensis RW3S1 - WLIP
Pseudomonas sp. RW10S2 - WLIP
P. farsensis
Pseudomonas sp. SWRI51
P. fakonensis COW40 - WLIP
P. xanthosomae COR54 - WLIP
P. sichuanensis
P. entomophila L48 - Entolysin
P. xantholysinigenes RW9S1A - Xantholysin
P. muyukensis COW39 - Xantholysin
P. maumuensis COW77 - Xantholysin
P. soli LMG 27941 - Xantholysin
P. peradeniyensis BW13M1 - Xantholysin
Pseudomonas sp. 250J - Xantholysin
Pseudomonas sp. BW16M2 - Xantholysin
P. mosselii BW18S1 - Xantholysin
P. mosselii 1A00316 - Xantholysin
P. mosselii
P. mosselii BW11M1 - Xantholysin
P. taiwanensis
P. promysalinigenes
P. putida AA7
P. vlassakiae RW4S2 - Putisolvin
P. putida W5
P. plecoglossicida
P. putida GM84
P. putida GIMC5401-PPKH-115
P. putida BR-PH17
P. putida W619
P. capeferrum WCS358 - Putisolvin
Pseudomonas sp. SWRI68 - Putisolvin
Pseudomonas sp. SWRI59 - Putisolvin
P. putida E41 - Putisolvin
P. putida E46 - Putisolvin
Pseudomonas sp. SWRI50 - Putisolvin
Pseudomonas sp. SWRI67 - Putisolvin
P. kermanshahensis SWRI100 - Putisolvin
P. fulva ATCC 31418 - Putisolvin
P. parafulva DSM 17004 - Putisolvin
P. jutendi
P. putida SY153
P. putida TJI-51
P. putida GB-1
P. putida PP112420
P. putida S13-1-2
P. putida
P. putida KF715
P. putida ZXPA-20
P. putida H8234
P. putida B1
P. putida R51
P. putida BS3701
P. alloputida
P. putida MX-2
P. putida LS46
P. putida 15420352
P. putida YC-AE1
P. putida T25-27
P. monteilii
P. monteilii 170620603RE
P. monteilii 170918607
P. monteilii STW0522-72
P. kurunegalensis
P. monteilii FDAARGOS171
P. oryzicola
P. anuradhapurensis
Pseudomonas sp. SK
P. persica
P. inefficax
P. shirazica
P. pyomelaminifaciens
P. asiatica
Subgroups
P. akappagea
P. japonica
P. vranovensis
P. cremoricolorata
P. reidholzensis
P. guariconensis
P. wayambapalatensis
P. farsensis
P. xanthosomae
P. mosselii
P. plecoglossicida
P. taiwanensis
P. vlassakiae
P. capeferrum
P. putida

Figure S5. Genome-based phylogeny of the *P. putida* group. The phylogenetic tree was inferred using IQ-TREE with automatic model selection (LG+F+R10) and 1,000 ultrafast bootstraps, based on an alignment of 2,570 orthogroups from 97 *Pseudomonas* strains (accession numbers Table S6). All strains included in this analysis, together with their accession numbers and the output of the prospection for CLP BGC are detailed in Table S6. Newly described species are highlighted in bold. The *P. rhizosphaerae* group are used as outgroup.
